# The Japanese guidelines for the management of sepsis

**DOI:** 10.1186/s40560-014-0055-2

**Published:** 2014-10-28

**Authors:** Shigeto Oda, Mayuki Aibiki, Toshiaki Ikeda, Hitoshi Imaizumi, Shigeatsu Endo, Ryoichi Ochiai, Joji Kotani, Nobuaki Shime, Osamu Nishida, Takayuki Noguchi, Naoyuki Matsuda, Hiroyuki Hirasawa, Sepsis Registry Committee of JSICM

**Affiliations:** Department of Emergency and Critical Care Medicine, Chiba University Graduate School of Medicine, 1-8-1 Inohana, Chuo-Ku, Chiba, 260-8677 Japan; Department of Emergency Medicine, Ehime University Graduate School of Medicine Shitsukawa, Toon, 791-0295 Japan; Division of Critical Care and Emergency Medicine, Tokyo Medical University Hachioji Medical Center, 1163 Tatemachi, Hachioji Tokyo, 193-0998 Japan; Department of Intensive Care Medicine, Sapporo Medical University School of Medicine, S1 W17, Chuo-ku, Sapporo, 060-8556 Japan; Department of Emergency Medicine, Iwate Medical University, 19-1 Uchimaru, Morioka, Iwate 020-0023 Japan; First Department of Anesthesia, Toho University School of Medicine, 6-11-1 Omori-nishi Ota-ku, Tokyo, 143-8541 Japan; Department of Emergency, Disaster and Critical Care Medicine, Hyogo College of Medicine, 1-1 Mukogawacho, Nishinomiya, 663-8131 Japan; Division of Intensive Care Unit, University Hospital, Kyoto Prefectural University of Medicine, Kyoto, 602-8566 Japan; Department of Anesthesiology and Critical Care Medicine, Fujita Health University School of Medicine, 1-98 Dengakugakubo, Toyoake, 470-1192 Japan; Department of Anesthesiology and Intensive Care Medicine, Oita University School of Medicine, 1-1 Idaigaoka Hazamacho, Yufu Oita, 879-5593 Japan; Emergency and Critical Care Medicine, Graduate School of Medicine Nagoya University, 65 Tsurumai-cho Showa-ku, Nagoya, 466-8550 Japan; Chiba University, 1-8-1 Inohana, Chuo-Ku, Chiba, 260-8677 Japan

## Abstract

This is a guideline for the management of sepsis, developed by the Sepsis Registry Committee of The Japanese Society of Intensive Care Medicine (JSICM) launched in March 2007. This guideline was developed on the basis of evidence-based medicine and focuses on unique treatments in Japan that have not been included in the Surviving Sepsis Campaign guidelines (SSCG), as well as treatments that are viewed differently in Japan and in Western countries. Although the methods in this guideline conform to the 2008 SSCG, the Japanese literature and the results of the Sepsis Registry Survey, which was performed twice by the Sepsis Registry Committee in intensive care units (ICUs) registered with JSICM, are also referred. This is the first and original guideline for sepsis in Japan and is expected to be properly used in daily clinical practice.

This article is translated from Japanese, originally published as “The Japanese Guidelines for the Management of Sepsis” in the Journal of the Japanese Society of Intensive Care Medicine (J Jpn Soc Intensive Care Med), 2013; 20:124–73. The original work is at http://dx.doi.org/10.3918/jsicm.20.124.

**Electronic supplementary material:**

The online version of this article (doi:10.1186/s40560-014-0055-2) contains supplementary material, which is available to authorized users.

## Sepsis Registry Committee, Japanese Society of Intensive Care Medicine

Chairman:

Shigeto Oda (Department of Emergency and Critical Care Medicine, Chiba University Graduate School of Medicine)

Committee members:

Mayuki Aibiki (Department of Emergency Medicine, Ehime University Graduate School of Medicine)

Toshiaki Ikeda (Division of Critical Care and Emergency Medicine, Tokyo Medical University Hachioji Medical Center)

Hitoshi Imaizumi (Department of Intensive Care Medicine, Sapporo Medical University School of Medicine)

Shigeatsu Endo (Department of Emergency Medicine, Iwate Medical University)

Ryoichi Ochiai (First Department of Anesthesia, Toho University School of Medicine)

Joji Kotani (Department of Emergency, Disaster and Critical Care Medicine, Hyogo College of Medicine)

Nobuaki Shime (Division of Intensive Care Unit, University Hospital, Kyoto Prefectural University of Medicine)

Osamu Nishida (Department of Anesthesiology and Critical Care Medicine, Fujita Health University School of Medicine)

Takayuki Noguchi (Department of Anesthesiology and Intensive Care Medicine, Oita University School of Medicine)

Naoyuki Matsuda (Emergency and Critical Care Medicine, Graduate School of Medicine Nagoya University)

Advisor:

Hiroyuki Hirasawa (Professor Emeritus, Chiba University), hhirasawa@faculty.chiba-u.jp

## Working group members for guideline development

Shinji Akitomi (Department of Emergency Medicine, Iwate Medical University)

Kazuaki Atagi (Division of Intensive Care Unit, Osaka City General Hospital)

Yoshito Ujike (Department of Emergency and Critical Care Medicine, Okayama University Graduate School of Medicine)

Moritoki Egi (Division of Intensive Care Unit, Okayama University Hospital)

Yasuyuki Kakihana (Department of Emergency and Intensive Care Medicine, Kagoshima University Graduate School of Medical and Dental Sciences)

Koji Goto (Department of Anesthesiology and Intensive Care Medicine, Oita University School of Medicine)

Teruo Sakamoto (Department of Emergency and Critical Care Medicine, Kurume University School of Medicine)

Jun-ichi Sasaki (Department of Emergency and Critical Care Medicine, Keio University School of Medicine)

Tomohito Sadahiro (Department of Emergency and Critical Care Medicine, Chiba University Graduate School of Medicine)

Norio Satoh (Department of Primary Care and Emergency Medicine, Kyoto University School of Medicine)

Junpei Shibata (Department of Anesthesiology and Critical Care Medicine, Fujita Health University School of Medicine)

Yasushi Suzuki (Department of Emergency Medicine, Iwate Medical University)

Hiroomi Tatsumi (Department of Intensive Care Medicine, Sapporo Medical University School of Medicine)

Hajime Nakae (Department of Emergency and Critical Care Medicine, Akita University School of Medicine)

Tomoyuki Nakamura (Department of Anesthesiology and Critical Care Medicine, Fujita Health University School of Medicine)

Masataka Nakamura (Department of Emergency and Critical Care Medicine, Chiba University Graduate School of Medicine)

Shin Nunomiya (Division of Intensive Care, Department of Anesthesiology and Intensive Care Medicine, Jichi Medical University)

Ryuichi Hasegawa (Division of Emergency Medicine, Tosei General Hospital)

Yoshiro Hayashi (Department of Intensive Care Medicine, Royal Brisbane and Women’s Hospital; UQ Centre for Clinical Research, The University of Queensland)

Seitaro Fujishima (Department of Emergency and Critical Care Medicine, Keio University School of Medicine)

Yoshiki Masuda (Department of Intensive Care Medicine, Sapporo Medical University School of Medicine)

Ken-ichi Matsuda (Department of Emergency and Critical Care Medicine, Yamanashi University School of Medicine)

Toshihiko Mayumi (Department of Emergency Medicine, Ichinomiya Municipal Hospital)

Naoya Yama (Department of Emergency, Advanced Critical Care and Emergency Center, Sapporo Medical University Hospital)

## Table of contents

I. Introduction
II. Methods
III. Diagnosis and treatment against infection
  1. Definition and diagnosis of sepsis
  2. Diagnosis of infection
  3. Antimicrobial therapy
  4. Imaging studies
IV. General management and supportive therapy
  5. Initial resuscitation and vasoactive drugs
  6. Ventilatory support
  7. Glycemic control
  8. Nutritional management
  9. Steroid
  10. Disseminated intravascular coagulation (DIC) treatment
  11. Acute blood purification
  12. Immunoglobulin
  13. Protease inhibitor
V. Summary

## Introduction

The Surviving Sepsis Campaign guidelines (SSCG) have attracted great attention in critical care medicine and are the most frequently applied guidelines in clinical settings. The initial version [1] was published in 2004, and a revised version [2] was developed and published in 2008, although very far behind schedule. Currently, the second revision was completed and published at the annual meeting of the Society of Critical Care Medicine (SCCM) in 2012. The Japanese Society of Intensive Care Medicine (JSICM) was part of the committee established for the development of the revised version of 2008.

The SSCG present various recommendations on diagnostic methods, management, and therapy associated with severe sepsis and septic shock, on the basis of the results of randomized controlled trials (RCTs) conducted in Europe and the United States. Previous reports have pointed out problems in the acceptance of the SSCG without any critical appraisal for clinical application in Japan [3],[4]. The major reasons are that the presence of genetic polymorphisms has a large influence on innate immunity and cytokine production, and racial differences in the distribution of such genetic polymorphisms cause the problem that therapies demonstrated to be efficacious in RCTs conducted in Europe and the United States are not always efficacious in Japan. However, it is also true that many RCTs from Europe and the United States had to be adopted in the development of this guideline.

The SSCG accept only treatments whose usefulness and efficacy have been confirmed by performing RCT. The SSCG do not refer at all to methods whose reported efficacy is based from experiences in Japan, for example, a method to remove various humoral mediators, which is pathophysiologically critical in sepsis, by continuous hemodiafiltration. Although treatment of severe sepsis and septic shock with awareness of measures against pro-inflammatory cytokines and high mobility group box 1 (HMGB-1) proteins is common in Japan, such a method is not widely used in Europe and the United States. Additionally, questions were raised about performing RCTs for many therapies in the area of critical care [5]. With such a background, we consider it significant to develop Japan’s version of the SSCG. For this purpose, we initiated the Sepsis Registry Committee of the JSICM to consider the practice of severe sepsis/septic shock management in Japan in the development of the guideline.

Some issues emerged in the development of Japan’s version of the guideline. Here, we describe the general points, whereas individual points are mentioned with each item in the guideline. For the title of this guideline, it became an issue among doctors whether to adopt *Haiketsusho* (*Sepsis* in Kanji) *Treatment Guideline* or *Sepusisu* (*Sepsis* in Katakana) *Treatment Guideline*. The current definition of sepsis is “infection-induced systemic inflammatory response syndrome (SIRS)”. Moreover, the severe forms are severe sepsis and septic shock. However, since this definition had been proposed, it has been pointed out to be different from the definition of sepsis that clinicians generally envisage from their experience [6]. The second SSCG revision committee argued to change the previous use of “severe sepsis/septic shock” to “sepsis”; however, no conclusion has been reached. Sepsis, as appropriate for critical care, was deemed “severe sepsis” in the second revised version, and the title of the guideline was changed accordingly. With such a historical background, the title *Haiketsusho* (*Sepsis* in Kanji) was adopted for this guideline. This guideline is the first sepsis treatment guideline in Japan. It is expected to increase the completeness and widely contribute to clinical practice along with the further investigation referencing the opinions of other academic societies in the future.

This guideline was prepared with adult septic patients in mind; therefore, in pediatric patients with sepsis, a pediatric intensivist or pediatric physician specialized in infectious diseases should be consulted before deciding whether to apply this guideline.

## Methods

The working groups for guideline development consisted of members of the JSICM, mainly members of the Sepsis Registry Committee, with one group for each item of the guideline. The guideline was prepared by the working groups through systematic retrieval, collection, and evaluation of the literature to extract evidence more objectively.

Each working group systematically retrieved the literature with a focus on the concept of evidence-based medicine. In the case of evidence not being found, the results of surveillances performed by the Sepsis Registry Committee of the JSICM were referred for the development of the guideline. (In the first surveillance, from October 1, 2007 to December 31, 2007, 47 institutions participated and 226 cases were subjected to analysis. In the second surveillance, from October 1, 2009 to March 31, 2010, 39 institutions participated, and 310 cases were included in the analysis).

This guideline was finally determined on the basis of evidence from the literature in addition to expert opinion mainly from the committee members.

### Literature retrieval method

In principle, systematic and comprehensive retrievals were performed of literature published not earlier than 2000. The databases searched were PubMed, MEDLINE (OVID), and Cochrane Database of Systematic Reviews by using the keywords “sepsis”, “severe sepsis”, and “septic shock”. In the Japanese literature, “Ichushi (Web)” was searched and the keywords “Haiketsusho”, “jusho Haiketsusho”, and “haiketsushousei shock” were used. RCTs, meta-analysis of RCTs, and other non-RCT literature when RCT was not existed were selected.

The reports retrieved above multiplied by keywords for each clinical question (CQ) prepared for the 13 items of the guideline were also retrieved. Three to five committee members for each item assessed the titles and abstracts of the retrieved studies by using the above method.

### Evidence level of literature

The evidence level of the literature was determined according to Additional file 1: Table II-1. It should be noted that in conditions associated with a high frequency of sepsis, for those with only clinical studies in which the cause of disease is diverse and unidentifiable, the results were described as having such background; moreover, it was noted that the description is not intended to be specific to sepsis depending on the CQ. The 2008 SSCG [2],[7],[8] and the “Nutritional management guideline for acute respiratory failure patients with ventilatory support,” developed by the Japan Society of Respiratory Care Medicine [9], were also referred.

Evidence accumulated from large-scale RCTs with ≥100 subjects or cohort studies with ≥1,000 subjects was considered to be high-quality evidence. Low-quality cohort studies or low-quality case–control studies refer to studies without explicit control groups, those that failed to evaluate exposure and outcome with the same objective method (blinding is desirable) for the exposure and nonexposure groups, those that failed to identify or appropriately control known confounding factors, and those that failed to perform complete follow-up for a sufficient period.

In principle, RCTs and meta-analyses were referred; however, if a paucity of evidence was found, the results of the investigation by the Sepsis Registry Committee were referred, and this fact was noted.

### Determination of the quality of recommendations

The GRADE of recommendations was determined according to the 2008 SSCG; however, this means the quality, but not the strength, of the recommendation (Additional file 1: Table II-2).

### Expression of the strength of recommendation

The strength of the recommendation was expressed by using two categories, as shown in Additional file 1: Table II-3, and additionally determined considering the expert opinion of committee members with reference to Additional file 1: Table II-4. The answer (A) for each CQ was expressed by using the strength of recommendation (1 or 2) added with the recommendation GRADE (A to D).

Even for high-quality studies, if the focus of the study was not limited to sepsis and such study only exists as evidence, the results were described as having been obtained from such a background, and an asterisk (*) was added to the evidence level. The recommendation was made, taking into account that the evidence was not specific for sepsis.

## Diagnosis and treatment against infection

### Definition and diagnosis of sepsis

CQ1: What is the definition of sepsis?

A1:

- *Haiketsusho* (in Japanese) = sepsis. Here, sepsis is defined as SIRS caused by infection, i.e., infection-induced SIRS.
- The definition of SIRS consists of any two or more of the following (1C):
- Body temperature >38°C or <36°C
- Heart rate >90 beats/min
- Respiratory rate >20 breaths/min or PaCO_2_ < 32 Torr
- White blood cell count >12,000 or <4,000 mm^3^ or immature granulocytes (band cells) >10%
- Pathogenic microorganisms need not be detected in blood culture (bacteremia), or toxins of bacteria need not be detected in blood (endotoxemia, etc.) (1C).
- The existence of infection is confirmed by demonstration of the presence of pathogenic or potentially pathogenic microorganisms, or toxins of those microorganisms, in normally aseptic tissue, fluids, or cavities; however, if sepsis as a systemic inflammatory response to infection is strongly suspected, this is treated as an infection even without the demonstration of pathogenic microorganisms in aseptic sites. Refer to the supplemental signs and variables in Additional file 1: Table III-1-1 to determine the diagnosis of sepsis (1C).

Comment: The definition of sepsis based on the concept of sepsis syndrome [10], advocated by Bone et al. [10], was proposed in the consensus conference of the American College of Chest Physicians (ACCP)/SCCM in 1991 and published in 1992 [11]. These diagnostic criteria were internationally widely recognized and later widely used in many clinical trials and other studies. However, because the definition of SIRS used in this definition was not specific, and the inflammatory response to infection cannot be accurately diagnosed, this definition was reevaluated in the SCCM/European Society of Intensive Care Medicine (ESICM)/ACCP/American Thoracic Society (ATS)/Surgical Infection Society (SIS) joint conference held 10 years later, and a new definition was published in 2003 [12]. The basic concept of sepsis as infection-induced SIRS was considered to remain valid, whereas some supplementary indices to diagnose sepsis were added, such as inflammatory response, clinical signs, and laboratory tests. However, the supplementary indices presented at that time included those that are inappropriate for actual clinical settings or those that have uncertain utility; accordingly, this guideline shows clinically useful indices.

Weiss et al. reported the results of the comparison of new and old diagnostic criteria in the same patient groups, in which no differences were found in morbidity and mortality as a whole [13]. The clinical usefulness of the new diagnostic criteria published in 2003 has not been reported thus far. It is difficult to say that these new diagnostic criteria have been widely used, probably because they are very complicated. Rather, the definition reported in 1992 tended to be used more frequently.

The definition of infection itself was limited to “pathological process due to invasion of pathogenic or potentially pathogenic microorganisms into normally aseptic tissue, fluid, or space” in the 1992 report. Later, pathophysiological elucidation proved that there is a risk of septic conditions even if bacterial invasion into aseptic sites is not found. (For example, the large intestine is not aseptic from the beginning of enteritis caused by *Clostridium difficile*. Furthermore, there is a risk of septic shock due to the toxin produced by the bacteria rather than due to the bacteria themselves). Therefore, the 2003 definition noted that “it is included in the definition of infection if sepsis as a systemic inflammatory response to infection is strongly suspected, even without a demonstration of pathogenic microorganisms in aseptic sites.” However, other definitions of infection exists, including that of the Centers for Disease Control and Prevention (CDC) [14] and that of the International Sepsis Forum (ISF) [15]; thus, currently, there is no unified consensus.

CQ2: Is severe sepsis/septic shock used as a sepsis severity category?

A2:

- Severe sepsis/septic shock is used as sepsis severity category (1C).
- Severe sepsis is a condition that presents organ dysfunction, decreased organ perfusion, or hypotension among septic patients. (Decreased organ perfusion or abnormal organ perfusion includes lactic acidosis, oliguria, and altered mental status). The organ dysfunction index for the multiple organ dysfunction score (MODS) score or the sequential organ failure assessment (SOFA) score is used to determine organ dysfunction (1C).
- Septic shock indicates a severe sepsis presenting hypotension (systolic blood pressure <90 or >40 mmHg decrease from the normal level) despite an adequate fluid challenge. However, if vasopressor is administered, hypotension may not occur (1C).

Comment: After the definition of severe sepsis/septic shock in the joint conference in 1991, many large-scale epidemiologic studies revealed that these criteria represented sepsis severity [16],[17]. There was no major change in the definition during the review in 2003, and the definition of organ dysfunction, which had remained vague, was decided to be applicable to the MODS score proposed by Marshall et al. [18] or the SOFA score proposed by the ESICM [19]. Although no report comparing this sepsis severity category with other clinical stratification was found, this sepsis severity category was used in various studies on sepsis treatment, including studies on early goal-directed therapy (EGDT) by Rivers et al. [20], administration of activated protein C by Bernard et al. [21], and small-dose steroid therapy by Annane et al. [22]. Therefore, there is sufficient basis for continuing use of this definition.

CQ3: Is there any useful biomarker for sepsis diagnosis?

A3: C-reactive protein (CRP), interleukin-6 (IL-6), and procalcitonin (PCT) may be useful as supplementary indices for sepsis diagnosis to some extent; however, there is currently no established biomarker for sepsis diagnosis (1C).

Comment: More than 170 biomarkers for sepsis have been examined [23],[24]. For application in clinical settings, a biomarker should meet the following criteria: high sensitivity/specificity for sepsis, easy to measure, provides rapid results, and inexpensive; however, no biomarker with these characteristics has been found thus far. CRP is widely used as an inflammatory biomarker; however, it lacks specificity for sepsis because its level increases in conditions other than infection. IL-6 is one of the inflammatory cytokines. IL-6 reflects the degree of hypercytokinemia, which causes systemic inflammatory response. IL-6 reaches a peak about 6 h after an insult. CRP and PCT are produced by the stimulation of IL-6; therefore, their peaks are delayed by 24–48 h after the peak of IL-6. The measurement of IL-6 enables early diagnosis of SIRS. Furthermore, IL-6 has been reported to reflect the severity and outcome in patients with sepsis [25]-[27].

PCT has been highlighted as the most promising biomarker; however, thus far, its usefulness has been reported mainly from Europe [28]. PCT measurement was also approved by health insurance in Japan from 2006. However, PCT is known to increase in noninfectious inflammation, including postsurgery or posttrauma; thus, its value for the diagnosis of sepsis is inconsistent [29]. The current cutoff values for CRP, IL-6, and PCT are presented in Additional file 1: Table III-1-1 as references for sepsis diagnosis. However, these values vary with different measurement methods, and therefore, the results should be interpreted with caution. IL-6 measurement is not yet covered by health insurance in Japan.

Measurement of endotoxin is theoretically useful for diagnosing gram-negative bacterial infection; however, no measurement system for accurate detection has been established yet. Mainly turbidimetric time assay in Japan and endotoxin activity assay with Food and Drug Administration approval in Europe and the United States are used as endotoxin measurement methods. Both methods were reported to produce results that correlate with the severity of sepsis [30]; however, their sensitivity and specificity are far insufficient for the diagnosis of sepsis. However, current efforts to study the combination of several biomarkers may lead to the discovery of a useful biomarker for the diagnosis of sepsis.

### Abbreviations:

ESICM: European Society of Intensive Care Medicine

ATS: American Thoracic Society

SIS: Surgical Infection Society

CDC: Center for Disease Control and Prevention

MODS: multiple organ dysfunction score

SOFA: sequential organ failure assessment

### Diagnosis of infection

CQ1: How should microbiological specimens be sampled?

A1:

- We recommend obtaining blood culture samples before starting antimicrobial administration (1D).
- We recommend obtaining specimen samples from the suspected infectious source aseptically and immediately send them for Gram staining, culturing, and antimicrobial sensitivity testing (1D).

Comment: As patients with severe sepsis/septic shock have a high possibility of having bacteremia, blood culture to determine the causative microorganism should be performed before empirical antimicrobials are administered [31]. Cerebrospinal fluid should be obtained in cases of suspected meningitis after excluding the possibility of intracranial hypertension. Bronchoscopic or blind bronchoalveolar lavage fluid samples should be obtained in cases of suspected ventilator-associated pneumonia for quantitative culture [32]. Tracheal aspiration might be acceptable if a patient has neither previous antimicrobial therapy nor risks of harboring antimicrobial-resistant pathogens [33],[34]. In cases of suspected central venous catheter-associated bacteremia, we recommend obtaining two sets of blood culture samples, one set from the indwelling catheter and another set from the catheter tip [35]. Any sampling should be performed before, but without delaying the initiation of, antimicrobial administration. We recommend performing Gram staining as a cheap and rapid diagnostic test.

CQ2: How should blood cultures be sampled?

A2:

- We recommend disinfecting the skin of the puncture site with alcohol-based chlorhexidine, alcohol-based 10% povidone-iodine, or alcohol followed by water-based 10% povidone-iodine (1B).
- We recommend obtaining at least two sets of 20 mL blood samples (at least three sets if infective endocarditis is suspected) through percutaneous puncture (1C).

Comment: Health-care personnel who will perform the sampling should perform hand washing and wear sterile gloves. The skin should be sufficiently disinfected before puncture. The contamination rate was found to be significantly lower when using 2% chlorhexidine or 0.5% chlorhexidine containing alcohol rather than aqueous povidone-iodine [36]-[38]. A chlorhexidine preparation is recommended as the primary disinfectant [35]. Japanese clinicians, however, might consider that 0.5% chlorhexidine solution is popular and 1% formulation is rare in Japan. No comparative studies between alcohol/chlorhexidine and alcohol/povidone-iodine exist [36]. We therefore suggest using 10% povidone-iodine (i) as an alcohol-containing preparation or (ii) in combination with pretreatment with alcohol, (iii) with sufficient waiting time after the application (i.e., until it dries up) to ensure efficacy.

In case of suspected catheter-related bloodstream infection, one set of blood samples should be drawn through the catheter. If endocarditis is suspected, more than three sets are recommended [39],[40]. The blood samples for each set should have a volume of 20 mL and be aliquoted into aerobic and anaerobic bottles [41]. The rubber top of the culture bottle should be disinfected with the same disinfectant used for the skin before injection. Other than fever, hypotension or shaking chills may suggest bacteremia, and therefore, blood culture should be performed.

After inoculation, the culture bottles should be kept at room temperature and placed in an incubator as soon as possible.

CQ3: What are the frequent sites of infection and causative microorganisms?

A3: The common infection sites are the intra-abdominal organs, respiratory tract, bloodstream (including catheter-related sites), skin/soft tissue, and urinary tract. The common causative organisms are *Staphylococcus aureus* (methicillin-resistant *Staphylococcus aureus* (MRSA), methicillin-susceptible *Staphylococcus aureus* (MSSA)), *Escherichia coli*, *Klebsiella pneumoniae*, *Pseudomonas aeruginosa*, *Enterobacter*, etc. (1C).

Comment: In a large-scale cohort study (EPIC II study) that investigated infections in the ICU, a 1-day prevalence study was performed in 1,265 ICUs worldwide, and the infection site, causative organisms, patient severity, and outcome were investigated in 7,087 patients with infection from a total of 13,796 cases [42]. The most common site of infection was the respiratory tract (63.5%), followed by the abdomen (19.6%), blood (15.1%), kidney/urinary tract (14.3%), skin (6.6%), catheter-related site (4.7%), central nervous system (2.9%), and others (7.6%) [42]. In another large-scale cohort study (Cooperative Antimicrobial Therapy of Septic Shock (CATSS) database study) in 5,715 patients with septic shock, pneumonia (37.2%) was the most frequent infection, followed by abdominal (30.1%), urogenital (10.9%), skin/soft tissue (8.1%), bloodstream (4.3%), catheter-related (3.4%), and central nervous system (1.1%) infections [43]. According to the first Sepsis Registry survey performed by the Sepsis Registry Committee of the JSICM, the frequent infections in 266 patients with severe sepsis were abdominal (32.0%), respiratory (25.9%), blood (15.8%), skin/soft tissue (10.2%), urinary tract (8.3%), and other (7.9%) infections [44].

The common causative organisms in ICU patients (EPIC II) were *S. aureus* (20.5%), *P. aeruginosa* (19.9%), *Candida* (17.0%), *E. coli* (16.0%), *K. pneumoniae* (12.7%), and *Staphylococcus epidermidis* (10.8%), and regional variations were also reported [42]. The common causative organisms in patients with septic shock (CATSS) were *E. coli* (24.6%), *S. aureus* (13.2%), *K. pneumoniae* (8.7%), *Streptococcus pneumoniae* (8.6%), *P. aeruginosa* (6.6%), fungi (6.5%), and group A *Streptococcus* (4.1%) [43]. From the result of the first Sepsis Registry survey, MRSA (22.0%) was the most frequent, followed by *E. coli* (14.0%), *K. pneumoniae* (11.8%), MSSA (9.7%), *P. aeruginosa* (9.2%), *Enterobacter* (7.4%), *S. pneumoniae* (6.0%), etc. [44].

The infections causing sepsis and the causative organisms vary depending on the country/region, hospital department, type of infection (community-acquired or nosocomial), and patient background. It is important to collect data from the clinician’s own facility as a reference and to consider the individual patient’s background [15].

### Antimicrobial therapy

CQ1: When should empirical therapy be started?

A1: Start empirical antimicrobial administration within 1 h after diagnosis (1C).

Comment: The second version of the SSCG recommends antimicrobial administration within 1 h after the diagnosis of sepsis, on the basis of a retrospective multicenter cohort study in patients with septic shock and a retrospective cohort study in patients with *Candida* bloodstream infections [45],[46]. New evidences have been published since then, and a large-scale prospective study in patients with septic shock showed higher mortality in a subgroup of patients in whom antimicrobials were administered later after the onset of shock [47]. With regard to the timing of antimicrobial administration, both a multicenter prospective observational study and a retrospective cohort study in patients with severe sepsis revealed that mortality tended to be low in proportion to the time between the recognition of sepsis and antimicrobial administration; in particular, mortality was significantly lower in a subgroup of patients in whom antimicrobials were administered within 1 h after the diagnosis of sepsis [48],[49]. In addition to candidal infection, as mentioned previously, a significant correlation between the delay in antimicrobial administration and higher mortality was shown in patients with *Acinetobacter* and pneumococcal infection [50],[51].

CQ2: Which antibiotics should be empirically chosen for severe sepsis and septic shock?

A2: To provide a successful empirical antimicrobial therapy, we recommend a broad-spectrum regimen with adequate coverage against typical causative organisms (1C) (Additional file 1: Table III-3-1).

Comment: To provide an appropriate empirical antimicrobial therapy, it is important to have an adequate microbiological differential diagnosis, with which the affected organ and place (i.e., community or hospital) where the septic episode occurred always need to be considered. The recommendable empirical antibiotic regimens for severe sepsis and septic shock are shown in Additional file 1: Table III-3-1. As inappropriate initial antibiotic therapy in severe sepsis and septic shock contributes to increased mortality, broad-spectrum antibiotic regimens should be employed to cover most of the typical organisms. As antibiograms vary depending on the time and place (e.g., country, region, institute, ward) [52],[53], each ICU should use regularly updated, unit-specific antibiograms.

The first national survey of severe sepsis and septic shock by the JSICM Sepsis Registry Committee revealed that MRSA and *P. aeruginosa* were the first and the fourth most frequent causative organisms of severe sepsis and septic shock, respectively, and these organisms are associated with higher mortality relative to the other organisms [44]. Therefore, special attention needs to be paid to whether or not treatment with the choice of initial antibiotic regimen should be given [54].

The association between the appropriateness of initial antibiotic therapy and mortality in sepsis has been reported in a number of observational studies. A recent meta-analysis that includes 70 studies confirmed this association [55]. A study in patients with MRSA bloodstream infection also showed a similar trend [56]. Therefore, broad-spectrum antibiotic regimens that cover most of the potential pathogens are recommended as empirical therapy for severe sepsis and septic shock.

On the other hand, to minimize antibiotic collateral damage, the use of unnecessary broad-spectrum antibiotics should be avoided. The emergence of carbapenem resistance in gram-negative bacilli (GNB) is a significant issue because loss of susceptibility to carbapenems often means the entire loss of a reliable and safe treatment option for serious GNB infections [57]. Prescription of carbapenems should be limited to situations in which these drugs are primarily required (Additional file 1: Table III-3-1).

Antibiotic combination therapy for severe sepsis and septic shock is a controversial issue. A recent RCT found no benefit of antibiotic combination therapy [58]. However, where nosocomial resistant GNB (especially *P. aeruginosa*) are of concern as causative organisms, the combination of an antipseudomonal beta-lactam (e.g., TAZ/PIPC, CFPM, MEPM, DRPM, IPM/CS) and an aminoglycoside is an acceptable option for increasing the chance of appropriate initial therapy [59],[60]. However, a recent observational study suggested a possible association between antibiotic combination therapy and deterioration of clinical outcomes [61]. Therefore, antibiotic combinations should be carefully employed, considering local antibiograms and the severity of the patient.

Addition of antifungal agents is another controversial issue in severe sepsis and septic shock. Whereas delayed initiation of antifungal therapy in candidemia is associated with poor mortality [44], the empirical use of antifungal agents did not demonstrate improvement in mortality [62]. An antifungal agent should be added when deep fungal infection or fungemia is clinically suspected, with careful risk assessment.

Complicated cases (e.g., immunocompromised patients, central nervous system infections, infective endocarditis, prosthetic device infections, osteomyelitis, unknown source of infection) require immediate consultation with infectious disease specialists after an initial dose of antibiotics. The lack of an infectious disease consultation service and shortage of infectious disease specialists are common issues in many hospitals in Japan. We suggest liaising with other hospitals and using Internet-based communication to overcome this issue.

CQ3: Which antibiotics should be chosen for a definitive antimicrobial therapy in severe sepsis and septic shock?

A3:

- Once a causative organism is identified, with the aid of susceptibility results, the initial antibiotic regimen should be changed to the most appropriate antibiotic agent (1D) (Additional file 1: Table III-3-2).
- Except for special situations, monotherapy should be employed for definitive antibiotic therapy in severe sepsis and septic shock (2B).
- In case of MRSA or *Candida* spp. bloodstream infection, consultation with infectious disease specialists is advisable (2D).

Comment: Once the causative organism is identified, the initially chosen antibiotic agents should be changed to an agent with more clinical evidence against the organism and has as narrow a spectrum as possible to minimize antibiotic collateral damages [63]. As a recent multicenter RCT showed similar clinical outcomes between meropenem monotherapy and the combination therapy of meropenem plus moxifloxacin [58], the benefit of combination therapy is unclear even in severe sepsis and septic shock. Thus, we recommend choosing monotherapy except for special situations in which clinicians believe monotherapy is inappropriate.

In cases where *Staphylococcus aureus*, *Enterococcus* spp., or *Candida* spp. are isolated from blood culture, infective endocarditis is suspected; a typical causative organism in infective endocarditis is isolated from blood culture; central nervous system infections, osteomyelitis, prosthetic devices, and immunosuppression (e.g., organ transplantation) are involved; and recommendable agents need to be avoided because of an allergy history. Consultation with infectious disease specialists is advisable.

CQ4: How should highly drug-resistant or multidrug-resistant organisms be treated?

A4: Consultation with infectious disease specialists is advisable because treatment for serious infections due to highly drug-resistant or multidrug-resistant organisms (Additional file 1: Table III-3-3) requires a high level of expertise (2D).

Comment: The treatment options for serious infections due to highly drug-resistant or multidrug-resistant organisms are limited, and no breakthrough drug is currently being developed [64]. Treatment of highly drug-resistant or multidrug-resistant gram-negative bacilli is especially challenging, and available treatment options are limited to high-dose administration of standard antibiotics or use of nonstandard antibiotics such as colistin [64].

MRSA is a common issue in Japanese ICUs. Although a few novel anti-MRSA agents such as linezolid and daptomycin have been shown to be noninferior to vancomycin in some disease conditions, vancomycin is still a drug of choice for most MRSA infections because of its higher clinical significance than novel agents. However, therapeutic drug monitoring (TDM) and dose adjustment based on TDM is required when using vancomycin to minimize its adverse effects such as nephrotoxicity. Recent observational studies have shown the association between vancomycin treatment for MRSA isolates with high vancomycin minimum inhibitory concentrations (MICs) and treatment failure. The use of novel anti-MRSA agents may be justified when MRSA with high vancomycin MICs are involved [65]-[67].

Daptomycin and linezolid have been shown to be noninferior to vancomycin for the treatment of skin and soft tissue infections and right-sided infective endocarditis, and skin and soft tissue infections and pneumonia, respectively [68]-[76]. Daptomycin and linezolid may be chosen for MRSA infections in selected situations for which clinicians believe these agents are more appropriate than vancomycin. However, these novel agents do not have sufficient clinical evidence for treating severe sepsis and septic shock, and the emergence of resistance to these agents and their adverse effects are of concern.

In the treatment of severe sepsis and septic shock due to highly drug-resistant or multidrug-resistant organisms, consultation with infectious disease specialists is advisable because treatment for such serious infections requires a high level of expertise. *In vitro* susceptibility testing results may not be reliable for selecting appropriate antibiotics. In addition, expert advice is required for the purpose of isolation precaution.

The recommendable treatment options for highly drug-resistant or multidrug-resistant organisms are shown in Additional file 1: Table III-3-3. Some of the recommended doses in the table are higher than the approved maximal doses in Japan. However, from the perspective of intensive care medicine and clinical infectious diseases, we believe that those higher doses are justifiable for saving critically ill patients with serious infections having no standard treatment options.

CQ5: How is the pharmacokinetics and pharmacodynamics (PK/PD) theory applied to the administration of antibiotics?

A5: Antibiotic administration based on the PK/PD theory is reasonable. The clinical efficacy of beta-lactams is dependent on the time above MIC (TAM), and that of aminoglycosides, fluoroquinolones, and glycopeptides is dependent on maximum concentration (C_max_) or area under the concentration curve per MIC (AUC/MIC) (1C*).

Comment: Antibiotic administration based on the PK/PD theory has the potential benefits of increased clinical efficacy and decreased adverse effects [64],[77]. The PD parameters of typical antibiotics associated with clinical efficacy are shown in Additional file 1: Table III-3-4. The approved doses of antibiotics in Japan are not always determined on the basis of the PK/PD theory. Some old antibiotics approved in the pre-PK/PD era are still being recommended in unreasonable doses. The recommendable doses of representative antibiotics in the ICU based on the PK/PD theory for severe sepsis and septic shock are shown in Additional file 1: Table III-3-5.

Extended infusion or continuous infusion of beta-lactams increases the TAM and tissue concentration of antibiotics and theoretically has further benefits in severe sepsis and septic shock [78]-[81]. However, at present, no RCT has been performed to assess the impact of those strategies on the clinically meaningful outcomes in severe sepsis and septic shock. Therefore, those strategies should be considered as optional strategies for highly drug-resistant or multidrug-resistant organisms.

CQ6: How should de-escalation therapy be performed?

A6: We recommend discontinuing the empirical antimicrobial therapy and initiating targeted therapy with antimicrobial agents with a narrower spectrum when causative organism(s) are identified and clinical improvement is obtained (de-escalation). We also recommend discontinuing any antimicrobial administration when the possibility of bacterial infection can be excluded (1C).

Comment: Excessive use of antimicrobial agents is associated with disadvantages such as disruption of the normal bacterial flora, selection of antibiotic-resistant pathogens, or higher cost. Thus, once the infecting organism(s) is detected and antimicrobial susceptibility is determined, the antimicrobial therapy should be changed to a targeted therapy with a single antimicrobial with a narrower spectrum (de-escalation). However, it should be considered that no RCTs have directly evaluated the direct effect of a de-escalation strategy on clinical outcome [82]. Cohort studies suggested that (i) when clinical response was favorable, early termination of antimicrobial administration was possible based on an early discontinuation policy in ventilator-associated pneumonia [83]; (ii) mortality is lower when de-escalation was successfully performed [84]; and (iii) de-escalation did not affect the relapse rate or mortality [85]-[87].

Clinicians should consider de-escalation in the following conditions:

1. Good quality of microbiological specimens is sampled before starting empirical therapy [88];
2. Clinical symptoms, including organ dysfunction and severity parameters, have improved;
3. The identified causative pathogen(s) is susceptible to narrower-spectrum antimicrobial agents;
4. Another focus of infection can be excluded; and
5. No significant immunodeficiency including continuous neutropenia (<1,000/mm^3^) exists.

CQ7: How is a decision to discontinue antibiotics made?

A7:

- A decision to discontinue antibiotics can be made when stabilization of vital signs and functional improvement of affected organs are achieved (1D).
- The standard duration of antibiotic treatment (Additional file 1: Table III-3-6) is recommendable in severe sepsis and septic shock caused by typical infections (1C).
- Procalcitonin-guided antibiotic cessation strategy may be appropriate (2A*).

Comment: The standard durations of antibiotic therapy shown in Additional file 1: Table III-3-6 are widely accepted by experts [89]. However, in patients with immunosuppression, central nervous infections, infective endocarditis, osteomyelitis, and bloodstream infection due to *S. aureus*, *Enterococcus* spp., and *Candida* spp., a high level of expertise is required to determine the sufficient antibiotic duration. Therefore, in those circumstances, consultation with infectious disease specialists is advisable.

Procalcitonin-guided antibiotic cessation policy has been studied in a few RCTs mainly in patients with respiratory tract infections and has been shown to decrease antibiotic duration without harming the patients [90],[91]. However, in severe sepsis and septic shock, this strategy has been tested in only one RCT with a small sample size [92]. Therefore, the use of procalcitonin-guided antibiotic cessation policy should be considered an optional strategy, and further clinical trials are required before its general use.

### Abbreviations:

AUC area under the plasma concentration time curve

C_max_maximum concentration

MIC minimum inhibitory concentration

MRSA methicillin-resistant *Staphylococcus aureus*

MSSA methicillin-susceptible *Staphylococcus aureus*

TAM time above minimum inhibitory concentration

TDM therapeutic drug monitoring

### Imaging studies

CQ1: When should imaging studies for detecting the source of infection be performed?

A1: For the purpose of source control and early administration of therapeutic strategies, identification/characterization of infectious loci should be performed as rapidly as possible after the initial resuscitation is done, if feasible (1C).

Comment: The SSCG recommend that imaging studies be performed promptly to identify the potential source of infection [2]. Early source control is preferable, and establishing a clinical diagnosis as soon as possible is a prerequisite [93]. Early source control including surgical procedures has been reported to be useful tools for better outcomes in patients with septic shock [94].

CQ2: Which imaging studies should be performed to identify a source of infection?

A2: In addition to bedside imaging techniques, such as plain X-ray and ultrasound, computed tomography (CT) is useful for screening the whole body to detect the source of infection if diagnosis is difficult (1D).

Comment: To detect the source of infection, performing enhanced CT is recommended. Chest CT has additional values/advantages over plain film radiography (PFR) in characterizing complicated pneumonia and detecting pulmonary complications [95],[96]. The imaging techniques recommended as useful tools for detecting various infectious diseases are summarized in Additional file 1: Table III-4-1.

CQ3: If enhanced CT could not detect any infectious lesions, what is next?

A3: Magnetic resonance imaging (MRI) can be considered an alternative approach. Consultation with a radiologist is favorable before performing MRI (2D).

Comment: As MRI can provide images with high resolution, it can detect meningitis [97], abscess, osteomyelitis [98], and wound infection [99] from various lesions (i.e., head, spine, and soft tissue)—conditions for which the detection ability of CT is limited. Radioisotope (RI) study to detect inflammatory foci may offer another possible alternative tool that can be used even in patients with chronic kidney disease, except in those pregnant or lactating. RI may be able to detect the source of infection when PFR, CT, and MRI have limited sensitivity and specificity [100].

## General management and supportive therapy

### Initial resuscitation and vasoactive drugs

CQ1: When should initial resuscitation be started?

A1: Initial resuscitation should be started when a progression of metabolic acidosis or an elevated blood lactate level is recognized irrespective of the presence of hypotension (1A).

Comment: Initial evaluation is performed for sepsis as infection-induced SIRS [101] according to item 1 (“Definition and diagnosis of sepsis”). Septic shock is a condition in which shock occurs with sepsis, and as the shock progresses, metabolic acidosis tends to progress and blood lactate level remains elevated.

Septic shock is defined to be present if the conditions are as follows: systolic blood pressure, <90 mmHg; mean arterial pressure (MAP), <60 mmHg; decrease in blood pressure of >40 mmHg from baseline without response to fluid resuscitation. Furthermore, the importance of the following was described in the joint conference of the SCCM/ESICM, ACCP, and ATS/SIS held in December 2001 [12]: hyperlactatemia (>1 mmoL/L, 9 mg/dL) and delay of capillary refilling time (>2 s) as an index of impaired tissue perfusion in sepsis.

The conference on the management of shock [102] held in April 2006 suggested that shock should not be diagnosed if the conditions are as follows: systolic blood pressure, <90 mmHg; mean blood pressure, <65 mmHg; and blood pressure reduction lower than baseline by >40 mmHg. Rather, it was recommended that evaluation of the progression of metabolic acidosis, elevated blood lactate level, and reduced central venous oxygen saturation (ScvO_2_) be performed. In addition, increase in blood lactate level [103]-[106], decrease in ScvO_2_[107],[108], and progression of metabolic acidosis [109] should be evaluated as indices of the progression of severe sepsis/septic shock, as suggested by many case reports and clinical studies.

CQ2: How is initial resuscitation monitored?

A2:

- Continuous monitoring of arterial pressure by using arterial line placement and arterial blood gas analysis in chronological order should be performed (1D).
- Initial resuscitation should target a central venous pressure (CVP) of 8–12 mmHg and an MAP of >65 mmHg mainly with fluid infusion, and it should be evaluated whether a urine volume of >0.5 mL kg^−1^ h^−1^ and ScvO_2_ of >70% were achieved (1A).
- Improvement of metabolic acidosis and lactate clearance should be evaluated by means of arterial blood gas analysis at least every 6 h (1A).
- Fluid management should be optimized by evaluating cardiac function and cardiac preload with echocardiography and other methods (2D).

Comment: In the initial resuscitation, monitoring by using an arterial line enables the continuous evaluation of blood pressure as well as blood sampling needed for arterial blood gas analysis in chronological order.

It should be evaluated whether a urine volume of >0.5 mL kg^−1^ h^−1^ and ScvO_2_ of >70% were achieved by initial resuscitation as an EGDT [20] with a goal of a CVP of 8–12 mmHg and an MAP of >65 mmHg. The improvement of survival rate in septic shock by using EGDT [20] was confirmed by some replication studies [110]-[112], including the first Sepsis Registry survey of the JSICM [44]. Also, improvement of metabolic acidosis and lactate clearance may be added in the evaluation of initial resuscitation [103]-[105],[113]-[115]. Infusion response and the proper infusion amount can be evaluated by performing echocardiography and other methods, including with the concomitant use of vasoactive drugs [116]-[119].

CQ3: How is initial resuscitation performed?

A3: Initial resuscitation is performed according to EGDT (1A). Crystalloid, as well as albumin and red blood cell (RBC) transfusion, is considered for initial infusion (2B).

Comment: An example of initial resuscitation for septic shock, which this guideline recommends, is shown in Additional file 1: Figure IV-5-1. It is recommended that initial resuscitation be performed according to EGDT [20]. For the infusion fluid, not only crystalloid solution but also albumin in combination with crystalloid may be used, according to the results of the SAFE study [120],[121]; however, this should be reevaluated in accordance with future large-scale clinical studies. In addition, the use of vasopressors (noradrenaline, vasopressin) is recommended in initial resuscitation for septic shock (see CQ4). Furthermore, RBC transfusion is recommended for anemia to maintain blood hemoglobin levels at >7 g/dL [2],[122].

CQ4. Which vasoactive drugs should be used for treatment of septic shock?

A4:

- For “warm shock,” characterized by warm peripheral temperature, at the early stage of septic shock, noradrenaline (~0.05 μg kg^−1^ min^−1^) is the first-choice drug (1A).
- If the responsiveness to noradrenaline is decreased, administering vasopressin (0.03 units/min) in combination to noradrenaline (~0.05 μg kg^−1^ min^−1^) could be considered (2B).

Comment: The early stage of septic shock is characterized by distributive shock with a decrease in systemic vascular resistance due to the production of vasodilating mediators. For this reason, noradrenaline alone or the combination of vasopressin and a small amount of noradrenaline is recommended as a vasopressor [123]-[127]. De Backer et al. [128] reported that dopamine increased the risk of the incidence of arrhythmia, including atrial fibrillation, in about a twofold higher rate than noradrenalin; therefore, the use of dopamine should be cautioned in patients with tachycardia or the risk of arrhythmia. In addition, dopamine also has a vasodilatory effect; thus, its superiority to noradrenaline is not obvious in the early stage of septic shock [129].

On the other hand, cardiac function is compromised from the early stage of septic shock owing to the effect of inflammatory cytokines and other factors, and it is difficult to improve cardiac performance with dopamine or dobutamine because of the impairment of intracellular signal transduction through the β1 adrenergic receptor [130],[131]. For this reason, to reduce the positive inotropic effect and the pulmonary artery pressure in case of cardiac dysfunction, it is preferable to consider the use of phosphodiesterase III inhibitors [132],[133] or calcium sensitizing agents [134] in combination with noradrenaline. In using these cardiac contractility-enhancing agents, assessment of cardiac function, such as with echocardiography, is required as a time series.

Furthermore, concerning the continuous administration of adrenaline in septic shock, sufficient international consensus has not been obtained yet. In the CAT study [135] comparing adrenalin and noradrenalin, there was no significant difference between the two groups in the median time to achieve an MAP of >70 mmHg; however, blood lactate and tachycardia were significantly increased in the adrenalin group. As this guideline recommends lactate clearance as one of the goal of initial resuscitation, adrenalin is not recommended at this time.

CQ5: What is the goal of initial resuscitation?

A5: The goal is to achieve an MAP of >65 mmHg, urine volume of >0.5 mL kg^−1^ h^−1^, ScvO_2_ of >70%, reduced blood lactate level, and improved metabolic acidosis within at least 6 h (1A).

Comment: The final goal of resuscitation from septic shock is improved blood lactate level and metabolic acidosis, which is an index of shock. This goal of initial resuscitation is based on EGDT [20]. Meanwhile, lactate clearance [(initial lactate − next measured lactate value)/initial lactate value × 100 (%)] is targeted at ≥10% at as long as 2 h after, and at ≥30% after 6 h [103]-[105],[113],[114],[136]. When the lactate clearance after 6 h is <10%, hypoperfusion of the hepatic or visceral area is suggested [137]. In patients with reduced renal function or acute kidney injury in whom a urine volume of >0.5 mL kg^−1^ h^−1^ could not be obtained for ≥6 h, diuresis may not be expected. It is preferable to consider increasing the blood pressure (MAP >80 mmHg) or the combination use of blood purification as renal replacement therapy.

### Ventilatory support

Mechanical ventilation during sepsis is considered an adjunctive therapy for secondary respiratory failure. However, no clinical study has been performed on respiratory failure caused by sepsis, and most clinical studies are limited to acute lung injury (ALI)/acute respiratory distress syndrome (ARDS). In fact, as statistical data show that >90% of patients with ALI/ARDS were complicated with sepsis, it is considered that creating this guideline on the basis of clinical data on ALI/ARDS could be valid [138].

Nevertheless, the clinical studies covered in this guideline are based on clinical data that have been accumulated according to the definition of ALI/ARDS established in 1994 [139]. More recently, the problem of this definition had been discussed, and the definition of ARDS has been revised according to the “Berlin definition” [140].

This item in the guideline covers clinical studies carried out on the basis of the previous definition. It is expected that treatment would be reevaluated more objectively when ARDS is studied with the new perspective of the Berlin definition in the future.

Although the ALI and ARDS cases following the 1994 definition are mixed in the clinical studies referenced here, a unified expression of ALI/ARDS was employed considering the “Berlin definition”.

CQ1: What is the target tidal volume?

A1: The target tidal volume is 6 mL/kg (expected standard body weight) (1A). Set the tidal volume to around 6 mL/kg (standard body weight) in the condition that the plateau pressure never exceeds >30 cm H_2_O (1A*).

Comment: There is no study that targeted only sepsis; however, lung compliance decreases in ALI/ARDS, requiring high alveolar pressure to provide an appropriate tidal volume and maintain PaCO_2_ within the reference range. However, patients present with extensive alveolar collapse and a small lung volume available for ventilation (the so-called baby lung condition) due to inflammation. If the patient is ventilated in such a lung condition with normal tidal volume or excessive alveolar pressure, the normal lung becomes impaired owing to the excessive ventilation volume and pressure. At the same time, it began to be pointed out from the late 1980s that induced shear stress in which the alveoli fallen into atelectasis due to inflammation repeatedly expand and collapse, consequently, are combined to cause ventilator-induced lung injury (VILI).

In the 1990s, four medium-scale RCTs on the influence of tidal volume on the prognosis of ALI/ARDS were conducted [141]-[144]. The results of three studies showed that large tidal volume itself does not affect the prognosis. On the other hand, the ARDS Network conducted a large-scale RCT in 2000 [145] in which one group was ventilated with a tidal volume of 6 mL/kg and alveolar pressure of not more than 30 cm H_2_O and the other group received a tidal volume of 12 mL/kg and alveolar pressure of not more than 50 cm H_2_O. This resulted in a 31% mortality rate in the first group, i.e., 9% better than that in the second group (see Additional file 1: Table IV-6-1).

The difference in the results of various RCTs, including the study of the ARDS Network, is considered to be due to the differences in tidal volume, alveolar pressure (plateau pressure), and positive endoexpiratory pressure (PEEP). Positive pressure ventilation by setting an appropriate tidal volume that never causes excessive alveolar pressure is required.

CQ2: What is the target of inspiratory plateau pressure?

A2: The higher the inspiration plateau pressure during mechanical ventilation, the worse the prognosis; however, the optimal level is difficult to determine (2B*).

Comment: There is no study that targeted only sepsis; however, a controlled inspiration plateau pressure of not exceeding 30 cm H_2_O was previously recommended. A recent meta-analysis demonstrated that a plateau pressure higher by 1 cm H_2_O causes a 1.03-fold increase in the odds ratio of mortality (95% CI, 1.01–1.06; *p* = 0.011); thus, a plateau pressure of 15–20 cm H_2_O leads to a 1.17-fold increase, 20–30 cm H_2_O to a 1.37-fold increase, and 30–50 cm H_2_O to a 1.87-fold increase in mortality [146]. Accordingly, maintaining the inspiration plateau pressure as low as possible is expected to contribute to improvement of outcome. However, it is shown that plateau pressure is inversely correlated with respiratory system compliance [146],[147], and the value varied with the condition of the lung and the PEEP level. In fact, plateau pressure reflects the impairment level of the lung and the plateau pressure within 48 h after the onset of ALI/ARDS is significantly associated with patient outcome, where the plateau pressure is measured after providing an appropriately low tidal volume and PEEP level.

The tidal volume in ALI/ARDS is usually targeted at 6 mL/kg; however, monitoring of the plateau pressure is expected to result in maintaining a lower tidal volume [146],[147]. On the other hand, the possibility of increased mortality is suggested because a collapsed lung cannot be recruited if the plateau pressure is low [148]; thus, the optimal control levels of plateau pressure, including tidal volume and PEEP, remain controversial.

CQ3: What is the target PEEP level?

A3: Use of appropriate PEEP level may prevent lung injury and improve outcome. However, a single optimal PEEP level is difficult to determine (1B*).

Comment: There is no study that targeted only sepsis; however, theoretically, in ventilatory support for ALI/ARDS, the application of appropriate PEEP may allow the extension of collapsed alveoli and improve oxygenation, resulting in the prevention of lung injury. Multiple large-scale RCTs that compared high PEEP with low PEEP were conducted previously [149]-[151], and some meta-analyses of these large-scale RCTs and additional small-scale RCTs were also reported [152]-[155]. The addition of PEEP of around 15 cm H_2_O in average showed the possibility of improved oxygenation and lowered mortality. Subanalyses suggested that patients with more severe lung injury obtained more benefit [152],[153].

An appropriate PEEP level is considered to be a level in which both the collapsed part and the excessively expanded part are minimal, and consequently the elastance of the whole lung is minimal [156],[157]. However, as the optimal level of PEEP is considered to vary with the degree of individual lung injury, it is difficult to determine a uniform and universal value.

In addition, there is a discussion in which priority is given to PEEP or F_I_O_2_ for the improvement of oxygenation, and a meta-analysis has reported a better prognosis when priority was given to PEEP [158]. However, as stated in the sections on plateau pressure (CQ2) and PEEP (CQ3), because the information of pressure depends on lung mechanics, evaluation should be based on elastance (compliance) and other factors; thus, a uniform answer could not be expected.

CQ4: What is the proper patient position during mechanical ventilation?

A4: The prone position should be considered in case of severe hypoxemia (PaO_2_/F_I_O_2_ < 100) (2C*).

Comment: There is no study that targeted only sepsis; however, any abnormality in ventilation-perfusion ratio is affected by gravity, and thus the prone position is presumed to be physically advantageous for gas exchange compared with the supine position. Ventilation in the prone position has already been pointed out to be useful in 1974 [159], and the potential benefit of the prone position compared with the supine position has been also reported in ALI/ARDS [160]-[163].

However, in many multicenter RCTs [164]-[166] and systematic reviews [167]-[171], overall improvement in mortality was not found in ALI/ARDS.

In 2010, Sud et al. published a systematic review of ten RCTs [172]. In this review, 919 patients ventilated in the prone position and 867 patients ventilated in the supine position were included in total; in the subgroup analysis of patients with severe hypoxia (PaO_2_/F_I_O_2_ < 100), 295 patients ventilated in the prone position and 260 patients ventilated in the supine position were included. As a result, the prone position was concluded to be superior to the supine position, with a risk ratio for death at 0.84 (95% CI, 0.74–0.96; *p* =0.01) in the severe hypoxemia group; however, the overall mortality was not significantly different. From this result, it is important to consider the prone position in ventilation for severe hypoxemia.

At the time of ventilation in the prone position, complications such as accidental removal of the catheter, tracheal tube, or central venous line; pressure sores; and ulceration of the face should be considered, with the understanding that these would require several resources, including manpower [167].

### Glycemic control

CQ1: How should the blood glucose level be targeted in patients with sepsis?

A1:

- A protocolized approach to blood glucose management in ICU patients with sepsis is recommended, commencing insulin dosing when blood glucose levels are >180 mg/dL (1A*).
- The target blood glucose level is set to 144–180 mg/dL (2A*), and intensive insulin therapy that maintains blood glucose level from 80 to 110 mg/dL should not be performed (1A*).

Comment: Intensive insulin therapy with target blood glucose levels of 80–110 mg/dL has been reported to reduce mortality in a single-center RCT in the cardiac surgery ICU [173]. Subsequently, an RCT performed in the medical ICU targeting patients with an estimated ICU stay of ≥3 days showed that intensive insulin therapy did not reduce overall mortality [174].

After the publication of the SSCG in 2008 [2], several RCTs [175]-[177] and meta-analyses [178],[179] on intensive insulin therapy have been reported. In these studies, intensive insulin therapy significantly increased the incidence of severe hypoglycemia (blood glucose level ≤40 mg/dL) [175]-[179]; however, mortality was not reduced [178],[177]. Also, intensive insulin therapy increased the 90-day mortality in the NICE-SUGAR trial [176]. Friedrich et al. reported in a meta-analysis that intensive insulin therapy is not beneficial for ICU patients both in surgical and medical ICUs [178].

The rationale for starting an insulin protocol with a blood glucose level of 180 mg/dL or higher and targeting a blood glucose level of 144–180 mg/dL is based on the NICE-SUGAR trial—the largest study among the RCTs that validated the target level of blood glucose in ICU patients. The American Diabetes Association and the American Heart Association both stated that the target level of blood glucose control should be set at 144–180 mg/dL for in-hospital patients [180],[181].

A subgroup analysis of the NICE-SUGAR trial demonstrated that there was no significant difference between nondiabetic patients and diabetic patients in the influence of intensive insulin therapy on mortality (odds ratio; nondiabetic patients vs. diabetic patients, 1.12 vs. 1.21; *p* = 0.60) [176]. Accordingly, the use of intensive insulin therapy cannot be recommended even for diabetic patients, and target blood glucose levels should be set to 144–180 mg/dL.

The DIGAMI study is a multicenter RCT comparing blood glucose control with target levels of <198 mg/dL and the control method without using insulin in post-myocardial infarction patients with an HbA1c of around 8% [182]. In the DIGAMI study, blood glucose control with target levels of <198 mg/dL significantly reduced the 1-year mortality compared with methods without insulin use. Diabetic patients have a high incidence of hypoglycemia [183],[184]; therefore, if patients with poor blood glucose control before becoming severe are judged to be at a high risk for hypoglycemia, the target of <198 mg/dL, which is higher than the target of 144–180 mg/dL, is acceptable.

Some foreign countries use the unit “mmol/L” for blood glucose level. As 1 mmol/L =18 mg/dL, the above values of 144, 180, and 198 mg/dL are calculated as 8, 10, and 11 mmol/L, respectively. As described below, errors in blood glucose measurement are relatively large, and hence, easier to measure values for clinical use, including 140–180 mg/dL and <200 mg/dL, can be used in performing blood glucose control.

Compared with conventional blood glucose control, blood glucose control by using an artificial pancreas in postoperative patients was reported to reduce the incidence of hypoglycemia, dose of insulin administered, days of hospital stay, and incidence of infection in single-center studies [185],[186]. However, no study has investigated the effectiveness of continuous blood glucose control by using an artificial pancreas in patients with sepsis.

CQ2: How should blood glucose level be measured in patients with sepsis?

A2:

- The blood glucose level of all patients receiving intravenous insulin therapy should be monitored every 1–2 h until the blood glucose level and insulin dose become stable, and every 4 h after stabilization (1C*).
- Blood glucose measurement by using capillary blood is not recommended because of large measurement errors and lack of accuracy (1B*).
- Blood glucose levels should be monitored by glucose meters or arterial blood gas analyzers using arterial/venous blood in patients with sepsis. When using these methods, the accuracy should be confirmed by performing blood glucose measurements at a central laboratory, as necessary (1B*).

Comment: Glucose meters are commonly used for blood glucose measurements in ICUs. However, blood glucose measurement by glucose meters using capillary blood are inaccurate and tend to be estimated higher, leading to inappropriate insulin infusion, then occurrence of hypoglycemia [187]. Blood glucose measurement by glucose meters using capillary blood are significantly inaccurate compared with those using whole blood or using a blood gas analyzer [187],[188]. In particular, in the hypoglycemic zone (blood glucose level of 72 mg/dL or lower), the error of glucose meter measurement with capillary blood is a major problem clinically; however, blood glucose measurement with a blood gas analyzer is more accurate [187]. The measurement error of blood glucose analysis is affected by various factors, including hematocrit and PaO_2_ of the sample blood, as well as drugs, blood sampling site, and type of measurement devices. In particular, patients with out-of-range blood glucose [187], those with anemia [188], hypotensive patients [188], and those required catecholamines [189] tend to have larger errors in blood glucose measurement.

Taking measurement time into account, blood glucose levels should be monitored by glucose meters or arterial blood gas analyzers using arterial/venous blood. However, these methods may also cause measurement errors; consequently, the accuracy should be confirmed by appropriately performing blood glucose measurements at a central laboratory.

### Nutritional management

CQ1: Should enteral nutrition be given priority over parenteral nutrition?

A1: Enteral nutrition should be given preferentially over parenteral nutrition (1B*).

Comment: Enteral nutrition, compared with parenteral nutrition, is thought to be effective in the maintenance of intestinal mucosa and in the prevention of bacterial translocation and organ dysfunction. There is no study that targeted only sepsis; however, many RCTs on trauma, burn, head injury, surgery, and acute pancreatitis reported that the incidence of infection [190], days of hospital stay, and health-care cost [191] were reduced by prioritizing the administration of enteral nutrition. Although the mortality was not decreased significantly [192], meta-analyses of these studies also demonstrated a reduction of the incidence of infection [192],[193] and days of hospital stay [193] by the preferential use of enteral nutrition.

In the first investigation of the Sepsis Registry Committee of the JSICM, the survival rate in the group with enteral nutrition was also significantly better than that in the group without enteral nutrition [44]. RCTs and meta-analyses in critically ill patients reported an improved patient outcome resulting from the preferential use of enteral nutrition rather than parenteral nutrition. Accordingly, it is strongly recommended to perform enteral nutrition rather than parenteral nutrition in patients with sepsis.

CQ2: What is the target calorie amount to be administered?

A2:

- The target calorie amount is calculated by using a convenient body weight conversion equation (25 kcal kg^−1^ day^−1^), a prediction formula for calculating calorie consumption, or measurement with indirect calorimetry (2D*).
- In obese patients (body mass index >30), measurement with indirect calorimetry or calculation based on ideal body weight should be performed (2D*).

Comment: As it has been reported that energy deficit, which is the difference between caloric need and the administered calories, was correlated with the number of complications in some prospective observational studies [194],[195], the target calories to be administered should be set and efforts should be made to achieve this target enterally.

There are >200 formulas for calculating the quantity of enteral nutrition; however, the optimal calculation method is unknown. The target level is set by using a convenient body weight conversion equation (25 kcal kg^−1^ day^−1^), a prediction formula for calculating calorie consumption (Harris-Benedict equation), or measurement of energy expenditure with an indirect calorimeter. Care must be taken when calculating the target calories for obese patients because the calculated calorie requirement may be overestimated if the prediction formula based on actual body weight is used.

CQ3: How should enteral nutrition be started?

A3:

- Enteral nutrition should be started within 24 h after admission or as early as possible (1B*).
- Use of vasoactive drugs is not a contraindication to early enteral nutrition; however, care should be taken when starting enteral nutrition in patients with unstable hemodynamics (1C*).
- It is not recommended to administer the full caloric needs from the beginning (1B*).

Comment: There is no clinical study comparing the early implementation of enteral nutrition with late implementation exclusively in patients with sepsis. Large-scale prospective observational studies in critically ill patients demonstrated that early enteral nutrition reduced mortality [196], infectious complications [197]-[199], days on mechanical ventilation [197],[200], and days of ICU stay [197],[200].

Meta-analyses of nutritional management in critically ill patients demonstrated that the introduction of enteral nutrition within 24 h resulted in significantly reduced [201],[202] or a downward trend of mortality [191], significantly decreased [201] or a downward trend of infectious complications [191], and reduced days of hospital stay [203].

Observational studies in patients receiving more than one vasoactive drug demonstrated that starting enteral nutrition within 48 h after the start of mechanical ventilation was associated with reduced mortality [204], and the use of vasoactive drugs was not a contraindication to the start of enteral nutrition. However, it should be noted that enteral nutrition in a condition where hypotension (MAP <60 mmHg) exists or the dose of vasoactive drugs has to be increased may cause ischemic enteritis [205].

From the above discussion, enteral nutrition is strongly recommended to be started within 24 h after admission or as early as possible. Also, the use of vasoactive drugs is not a contraindication to early enteral nutrition; however, it should be carefully started in patients with unstable hemodynamics.

There is no clinical study on the effect of enterally administered calories exclusively in patients with sepsis; the available studies involved ICU patients. In RCTs that compared the administration of full calories from the first day and progressive increase by starting with small amounts of enteral nutrition (providing 20% of the calorie requirement for 4 days [206] and increasing progressively starting with 10–15 mL/h [207],[208]), no difference in mortality between groups was found [206]-[208]. However, increased infectious complications [206],[208] and a trend of increased incidence of diarrhea and a significant increase in stomach residue [207] have been reported in the group started with full-calorie administration from the first day. From these results, full-dose enteral nutrition (i.e., equivalent to all caloric needs) is not recommended at the start of enteral nutrition. It is desirable to start from a small dose and progressively increase toward the target caloric level, while taking account of the regurgitated volume of enteral nutrition and the presence of diarrhea.

CQ4: What are the needs for supplemental parenteral nutrition during enteral nutrition?

A4: Unless malnutrition exists before a serious condition develops, calories should be provided mainly by enteral nutrition for 7 days after the onset of sepsis, and aggressive supplemental parenteral nutrition to achieve the target calories should not be provided (1B*).

Comment: There are no studies on the effect of supplemental parenteral nutrition only in patients with sepsis; many existing studies involved ICU patients. In a systematic review comparing studies on supplemental intravenous nutrition (i.e., supplementary parenteral nutrition is provided if the target calories are not achieved with enteral nutrition) and enteral nutrition alone [209], all five studies included in the review showed no differences in mortality, infections, days of hospital stay, and duration of mechanical ventilation. A large-scale RCT was reported in 2011 on the use of enteral nutrition to achieve the target calories, calculated by using a prediction formula with corrected ideal body weight (36 kcal kg^−1^ day^−1^ for men and 30 kcal kg^−1^ day^−1^ for women in those aged 60 years or younger; 30 kcal kg^−1^ day^−1^ for men and 24 kcal kg^−1^ day^−1^ for women in those aged 61 years or older). This study compared a group that received supplemental parenteral nutrition for the deficit from the target calories within 48 h and a group administered with only vitamins and trace elements for the initial 7 days and started with the supplemental parenteral nutrition on day 8 or later. The results showed a significant increase in the early survival discharge from the ICU and hospital, reduced incidence of infection, reduced number of patients needing mechanical ventilation for ≥2 days, reduced duration of renal replacement therapy, and reduced health-care costs in the group started on supplemental parenteral nutrition from day 8 [210]. The subgroup analysis in patients with sepsis showed similar results. The actual dose of enteral nutrition was 20–25 kcal kg^−1^ day^−1^ in this study.

Caloric intake through enteral nutrition is recommended in the initial 7 days for the treatment of sepsis in patients who have no malnutrition before developing a serious condition; however, supplemental parenteral nutrition aiming to achieve target calories is not recommended because there is a risk of a deteriorated prognosis. Vitamins and trace elements can be administered. The combined administration of enteral and supplemental parenteral nutrition to achieve the target calories is performed from day 8 and later.

CQ5: Is immunonutrition effective?

A5:

- There is no adequate data to recommend the enteral supplementation of glutamine (2B*).
- Administration of nutrition formulas containing arginine is not recommended for patients with severe sepsis (2B).
- Use of eicosapentaenoic acid (EPA)-, docosahexaenoic acid (DHA)-, γ-linolenic acid-, and antioxidant-enriched formula can be considered (2B).

Comment: Immunonutrition formulas (immune-enhanced nutrition formulas and immune-modulatory nutrition formulas) vary in nutrient contents depending on the product; therefore, it is difficult to compare immunonutrition formulas as a whole. Meta-analyses on immunonutrition formulas as a whole in critically ill patients did not demonstrate effectiveness [211],[212]. Additionally, there are few studies in patients with sepsis focusing on individual nutrients.

1. Glutamine
  There are few studies in patients with sepsis given an enteral dose of glutamine. Beale et al. compared a group that received nutrients containing 30 g glutamine + antioxidants administered from a nasogastric tube within 24 h, and subsequently received immunonutrition formulas within 48 h, and a control group among 55 patients with sepsis [213]. The glutamine group showed a significant reduction in the SOFA score compared with the control group; however, there was no difference in the final mortality rate. Schneider et al. reported that no difference was found in days of ICU stay, incidence of infection, and other parameters between a group that received nutrients containing 30 g glutamine + antioxidants and a control group among 58 critically ill patients (66% patients with sepsis and 34% patients with multiple trauma) [214]. In a meta-analysis of 31 studies, Avenell reported that data in ICU patients showed no improvement on mortality and incidence of infection by an enteral dose of glutamine [215]. Also, the topic of intravenous administration of glutamine is debated mainly in Europe, and the SIGNET trial conducted in ICUs at ten sites in Scotland showed no effects of glutamine (20.2 g/day) and selenium (500 μg/day) on infectious complications and mortality [216]. Currently, a 2 × 2 clinical study (REDOXS; http://clinicaltrials.gov/show/NCT00133978) of a group receiving both an enteral dose of glutamine (30 g/day) and intravenous administration (0.35 mg kg^−1^ day^−1^) vs. a control group and, further, an antioxidant group vs. a control group is being conducted in Canada, the United States, and Europe [217]. The study aims to enroll 1,200 patients and is expected to finish by the end of 2011, and the results may lead to a major evaluation of the effectiveness of glutamine. From the above discussion, data on glutamine administration in septic patients are insufficient to reach a conclusion.
2. Arginine
  Arginine improves immune function, enhances protein synthesis, and promotes wound healing, and is a substrate of nitric oxide production, which has an important role in the regulation of microcirculation. However, there are concerns that excessive production of nitric oxide may cause excessive dilation of peripheral vessels and have adverse effects on hemodynamics. Galbán et al. reported that mortality was significantly reduced in an arginine-enriched nutrient group compared with a control group in a study of 176 patients with sepsis (19% vs. 32%) [218]. On the other hand, Dent et al. reported that there was a significant increase in mortality in an arginine-enriched nutrient group compared with a control group in a study of 170 patients with sepsis (23% vs. 10%) [219]. Kieft et al. reported that there were no differences in mortality, incidence of infection, days of ICU stay, and other parameters between an arginine-enriched nutrient group and a control group in a study of 597 ICU patients [220]. There was no significant difference in mortality or incidence of infection in meta-analyses on the administration of arginine to patients with severe sepsis or critically ill patients except those with trauma and burns [213],[218]-[222]. From these results, the effect of administration of arginine-enriched nutrients under septic conditions has not been established, and it was weakly recommended that arginine-enriched nutrients should not be used for severe sepsis because arginine has been reported to worsen the condition.
3. EPA-, DHA-, γ-linolenic acid-, and antioxidant-enriched nutrients
  Three RCTs studied the effects of EPA-, DHA-, γ-linolenic acid-, and antioxidant-enriched nutrients containing 55% lipid (Oxepa®) in patients with sepsis [223]-[225]. Pontes-Arruda et al. reported that the use of this formula resulted in a significant improvement of oxygenation index, incidence of organ dysfunction, and survival rate, as well as decrease in days of mechanical ventilation and days of ICU stay in 165 patients with severe sepsis accompanied by ALI/ARDS, compared with a control formula with almost similar lipid contents [223]. However, the statistical method of this study was criticized because intention-to-treat analysis was not performed. Similar results were reported in a retrospective subgroup analysis of this study [223] on subjects in Brazil with Japanese ancestry [226]. Subsequently, Pontes-Arruda et al. studied the effect of Oxepa by using normal nutrients with 29% lipid content as a control in 106 patients with early-stage sepsis. Administration of enriched nutrients resulted in a significant reduction in the incidence of severe sepsis, new cardiovascular event, organ dysfunction such as respiratory failure, days of ICU stay, and days of hospital stay; however, there was no significant difference in mortality [224]. Grau-Carmona et al. compared Oxepa and normal nutrients with 30% lipid contents in 133 patients with sepsis [225]. The use of Oxepa increased mortality, although it was not statistically significant, whereas there was a significant reduction in days of ICU stay (16 days vs. 18 days). Furthermore, there was no difference in oxygenation index, incidence of infection, and incidence of organ dysfunction [225]. Rice et al. compared a group with enteral supplementation of EPA, DHA, γ-linolenic acid, and antioxidant administered intermittently with a nonadministered group (EDEN-OMEGA study) [227]. In this study, the group with enteral supplementation of EPA, DHA, γ-linolenic acid, and antioxidant administered intermittently demonstrated a significant increase in mortality compared with the nonadministered group. The EDEN-OMEGA study differs from the abovementioned three RCTs in the method of administration; that is, fish oil was directly administered intermittently in EDEN-OMEGA, whereas it was administered as an enteral formula continuously in the three RCTs. Therefore, the result of the EDEN-OMEGA study does not strongly deny the efficacy of Oxepa. From these results, the use of an enteral formula enriched with EPA, DHA, γ-linolenic acid, and antioxidants for patients with sepsis may contribute to the reduction of organ dysfunction, and thus, this was classified as a weak recommendation.

CQ6: What are the effects and evidences of selective digestive decontamination (SDD) and selective oropharynx decontamination (SOD)?

A6: SDD and SOD were reported to reduce mortality in patients who need intensive care. However, these methods are not recommended to be performed aggressively because their efficacy in carriers of drug-resistant bacteria is uncertain, and there is an increased possibility of the emergence of resistant bacteria (2B).

Comment: SDD is a method used to prevent the onset of hospital-acquired infections, including ventilator-associated pneumonia and bloodstream infection due to bacterial translocation, by administering nonabsorbable antimicrobials through the digestive tract and selectively suppressing the proliferation of aerobic gram-negative bacilli and fungi, which are the major causes of hospital infections. SDD was first reported by Stoutenbeek et al. in the Netherlands with effects on trauma patients [228]; thereafter, the effects of SDD together with its subtype, SOD, were reported in many RCTs and meta-analyses [229]-[232]. In a large-scale RCT performed in 5,939 ICU patients at 13 ICUs in the Netherlands in 2009, groups treated with SDD and SOD showed reduced mortality compared with the nonintervention group [233].

It is common to administer polymyxin plus aminoglycosides or a new quinolone against gram-negative bacteria, and in combination with amphotericin B against fungi; however, the appropriate drug types and dose for SDD are not established yet [234]. Also, the ineffectiveness of SDD against carriers of bacteria that that are resistant to SDD drugs (MRSA, vancomycin-resistant *Enterococcus*, extended-spectrum beta lactamase-producing gram-negative bacilli, etc.) and the fear about the emergence of new resistant bacteria by performing SDD have been pointed out as problems [229],[231],[235]-[237]. The use of SDD for patients with sepsis was about 3% in Japan (from the first investigation of the Sepsis Registry Committee).

An RCT at a single center reported that the use of SDD caused a significant increase in the detection rate of resistant gram-positive cocci in the intestine (17.0% vs. 80.7%, control vs. SDD) and also a significant increase in the detection rate of resistant coagulase-negative *Staphylococcus* (25% vs. 66.9%) [235]. A multicenter cohort study also reported an increased rate of detection in the intestine for resistant gram-negative bacteria (7% vs. 15%) [236]. Although the effectiveness of SDD and SOD is demonstrated in an RCT and meta-analysis, their effectiveness in carriers of resistant bacteria is uncertain. Consequently, it is weakly recommended not to perform SDD and SOD, owing to the possibility of an increase in resistant bacteria.

### Steroid

CQ1: What is the indication of steroid therapy in sepsis?

A1:

- The use of steroids is aimed at early recovery from shock in adult patients with septic shock who do not respond to initial fluid resuscitation and vasoactive drugs (2B).
- Adrenocorticotropic hormone (ACTH) testing is not required to determine the indication for steroid therapy (2B).
- Concerning the adverse effects of steroid therapy, it should be noted that the incidence of *de novo* sepsis and septic shock are significantly higher, other than hypernatremia and hyperglycemia (2B).

Comment: A multicenter RCT conducted in France, involving 300 adult patients with septic shock who did not respond to initial fluid resuscitation and vasoactive drugs [22], reported improved early recovery from shock and reduced 28-day mortality with steroid therapy in patients with relative adrenal insufficiency (defined as patients who had an increased cortisol level of <9 μg/dL at 30–60 min after the ACTH stimulation test). Subsequent small-scale RCTs [238],[239] also reported early recovery from shock. However, a large-scale multicenter RCT (CORTICUS study) involving 500 patients with septic shock in Europe in 2008 [240] showed that the time to reversal of shock was significantly shorter but the 28-day mortality was not reduced in the group with steroid therapy. In both the French trial and the CORTICUS study, low-dose and long-term administration of hydrocortisone at 200 mg/day for 5–7 days was employed, and the differences in results are considered to be due to the severity of the patients (mortality in the control group: 61% in the French trial vs. 31.5% in the CORTICUS study) and the delay in the start of steroid therapy (French trial: within 8 h after onset of shock vs. CORTICUS study: within 72 h). Recent meta-analyses [241],[242] concluded that low-dose and long-term steroid therapy (hydrocortisone at ≤300 mg/day for ≥5 days) resulted in early reversal of shock but no improvement in 28-day mortality.

Among 246 patients with sepsis enrolled in the first investigation of the Sepsis Registry Committee of the JSICM in 2007 [44], 63 matched pairs of patients with septic shock were compared by classifying them into two groups according to the use of steroid therapy. Matching was performed by using propensity scores with four background factors, including age, sex, APACHE II score, and SOFA score, and with eight laboratory data and treatment factors, including lactate measurement, blood culture before antimicrobial administration, antimicrobial administration within 1 h after ICU admission, and implementation of EGDT. There were no significant differences in the 28-day mortality and in-hospital mortality between the two groups [44]. Furthermore, none of the items in the treatment with sepsis bundle after steroid administration was significantly different between the two groups.

Although the efficacy of steroid was suggested to be higher in patients in whom cortisol was not increased by the ACTH stimulation test than in those with increased cortisol, subsequent studies failed to demonstrate a statistically significant association between the results of the ACTH stimulation test and the efficacy of steroids [22],[241], and recent multicenter RCTs also failed to obtain evidence [243]. The commonly used immunoassay for cortisol measures the total cortisol concentrations (protein-bound and free cortisol), in which free cortisol is the active fraction. The proportions of protein-bound and free cortisol vary in critically ill patients, and thus, the physiologically active free cortisol concentration cannot be measured accurately [243]. Because meta-analysis showed no differences in the efficacy of steroids, irrespective of the results of ACTH test, and taking into account the problem of cortisol measurement [244],[245], the ACTH stimulation test is not recommended for determining the indication for steroid therapy [241],[243].

Concerning the adverse effects, it should be noted that a significantly increased risk of severe infection such as *de novo* sepsis or septic shock has been pointed out other than the incidence of hyperglycemia and hypernatremia, even in low-dose and long-term steroid therapy. In addition, muscle weakness rarely occurs.

Consequently, although low-dose and long-term steroid therapy for septic shock causes early shock reversal, it does not improve the prognosis. Moreover, it should be used with caution, taking into consideration the occurrence of hyperglycemia, hypernatremia, and superinfection as adverse effects [2],[241],[243].

CQ2: When should steroid therapy be started?

A2: Steroids are administered at the early stage of shock onset (2C).

Comment: No RCT directly comparing the timing of the start of steroids with recovery rate from shock or 28-day mortality has been reported. Adult patients with septic shock who do not respond to initial fluid resuscitation and vasoactive drugs are targeted to receive steroid therapy. An RCT conducted in France [22] in which steroids were administered within 8 h after the onset of shock demonstrated better improvement not only in recovery from shock but also in mortality, compared with the CORTICUS study [240] in which steroids were administered within 72 h after the onset of shock. Thus, the usefulness of early administration of steroids is suggested.

CQ3: How should steroids be administered and what should be the duration of treatment?

A3:

- Low-dose and long-term steroid therapy, such as ≤300 mg/day hydrocortisone for ≥5 days, is recommended (1A).
- For hydrocortisone, an equivalent dose of 200 mg/day is divided into four doses, or a continuous infusion of 10 mg/h (240 mg/day) is administered after a bolus dose of 100 mg (2B).

Comment: Two RCTs and one meta-analysis concluded that high doses of corticosteroids for the treatment of severe sepsis or septic shock were ineffective, or even rather harmful [246],[247]. Large-scale RCTs on septic shock conducted by Annane et al. [22] and by Sprung et al. [240] used low-dose steroid, such as 200 mg/day hydrocortisone, divided into four doses. Early recovery from shock was obtained in both RCTs; Annane et al.’s study showed significant improvement in 28-day mortality, but Sprung et al.’s did not. A meta-analysis by Annane et al. [248] classified patients into four groups according to steroid dose and duration of treatment. The dose of steroids was classified into high and low doses, with the hydrocortisone dose of 300 mg/day as the border value, and the duration of treatment was classified into long-term and short-term treatments, with 5 days as the border value. According to these definitions, 17 RCTs were evaluated and improvement in the recovery rate from shock and in the 28-day mortality were demonstrated in the low-dose and long-term steroid therapy group. In a meta-analysis by Moran et al. [241] classifying patients into high-dose and low-dose groups, with 1,000 mg/day hydrocortisone as the border value, improved recovery rate from shock was found in the low-dose group.

From these results, although the effect of improving mortality is still controversial, low-dose and long-term steroid therapy with ≤300 mg/day hydrocortisone for ≥5 days is considered to be effective in obtaining early recovery from shock.

CQ4: What kind of steroid should be used?

A4: Hydrocortisone should be used (1A). Methylprednisolone can be used as an alternative (2C); however, dexamethasone or fludrocortisone should not be administered (2B).

Comment: Hydrocortisone, an endogenous steroid, is usually favored for use in patients with septic shock. However, there is no study comparing the effect and adverse effects according to the type of steroid. Meduri et al. recommended using methylpredonisolone, which has no mineralocorticoid effect and rarely causes electrolyte disorder, at 1 mg kg^−1^ day^−1^ for 14 days after a 1 mg/kg bolus administration for septic patients with ARDS [249]. Methylprednisolone, as the glucocorticoid, is given at five times the titer, 1.3 times the half-life, and one-half the dose of hydrocortisone [241]. Dexamethasone was often used until the introduction of the ACTH stimulation test; however, dexamethasone should not be administered because it has a long half-life and suppresses the hypothalamus-pituitary-adrenal axis immediately, and persistently, after administration [22],[238]. Also, an additional administration of fludrocortisone compared with that of hydrocortisone alone significantly increases infections, including urinary tract infections, and therefore should not be performed [250].

CQ5: How long should steroids be administered?

A5: Steroids should be gradually discontinued if administration of vasoactive drugs is no longer required (2D).

Comment: Concerning the methods for the administration of steroids, no study has compared between administration of the same dose throughout the dosing period and changing the dose on the basis of the clinical course. Moreover, no study has compared between gradual tapering and sudden drug discontinuation.

The same dose protocol was employed in three RCTs [22],[239],[240]. A gradual reduction of dose after the recovery from shock was done in two RCTs [238],[251]; steroids were administered for 2–5 days followed by a gradual decrease in dose for 2–14 days in four RCTs [238]-[240],[251], and steroids were suddenly discontinued after 7 and 10 days of administration in two RCTs [22],[252], respectively.

One crossover trial showed a rebound phenomenon in hemodynamics and immune function after the sudden discontinuation of steroid [253], whereas a tapering schedule of steroid did not reveal clear therapeutic effects.

### Disseminated intravascular coagulation (DIC) treatment

CQ1: Should DIC complicated with sepsis be treated?

A1: DIC in sepsis contributes to the development of organ failure; accordingly, treatments for DIC are suggested (1C*).

Comment: Sepsis has been reported to account for approximately 50% of all causes of DIC [254]. However, no study has evaluated treatments for DIC due to sepsis, although the 28-day mortality in severe sepsis has been reported [2]. Although results related to the 28-day mortality after anticoagulant administration in severe sepsis have been reported in the SSCG, treatment for DIC due to sepsis (septic DIC) has not been evaluated [2]. DIC is significant in sepsis because it can contribute to the development of organ failure. Triggered by infections, various cytokines are released from immunocompetent cells, including monocytes and vascular endothelial cells, followed by the activation of intravascular coagulation leading to intravascular thrombin formation. As a result, fibrinogen changes into fibrin, which is converted to fibrin polymers in the presence of coagulation factor XIII. Subsequently, generated fibrin clots adhere to platelets and red blood cells, leading to intravascular thrombosis. Eventually, the thrombi impair the microcirculation in each organ and induce multiple organ failure [255],[256]. Furthermore, thrombus formation is further promoted by increased plasminogen activator inhibitor-1 activity, which inhibits the fibrinolysis of the clots [257]. The abnormalities of the coagulation system in sepsis, as described above, are the theoretical background of anticoagulant therapy for septic DIC.

CQ2: How is septic DIC diagnosed?

A2: The Japanese Association for Acute Medicine (JAAM) DIC diagnostic criteria in critically ill patients are the most sensitive criteria and therefore recommended for the early diagnosis of septic DIC (1B*).

Comment: A DIC *ad hoc* committee of the JAAM published the acute-phase DIC criteria in 2005 as the more appropriate diagnostic criteria, especially for DIC due to emergency diseases, including infection, trauma, and burns. These acute-phase DIC diagnostic criteria compensate for the shortcomings of the DIC criteria advocated by the Ministry of Health, Labour and Welfare (MHLW) [258]-[260]. These diagnostic criteria enable the early diagnosis of DIC by using limited items measured in the off-hours at many institutions and have high sensitivity. Cases diagnosed as DIC by using these criteria were reported to have a mortality of about 20%–21% [258],[259],[261]; however, the mortality has been shown to vary depending on the primary disease of DIC as follows: 34.7% (34 of 98 patents) with sepsis and 14.8% (19 of 128 patients) with trauma, burns, or surgery [261].

The first Sepsis Registry survey by the JSICM [44] showed that 234 patients (88.0%) of the 266 registered patients with severe sepsis or septic shock were complicated with DIC according to the JAAM DIC diagnostic criteria, and 187 of 234 patients (79.9% of patients complicated with DIC) received certain DIC treatments.

CQ3: When should DIC treatments be initiated?

A3: DIC treatments should be started when DIC was diagnosed according to the JAAM DIC diagnostic criteria (2C*).

Comment: Before the JAAM DIC diagnostic criteria, the MHLW DIC criteria had been widely used; however, DIC treatments are known to be initiated even without fulfilling the diagnosis of DIC (>7 points) in clinical settings [262]. Also, a retrospective study [263] reported that the higher the DIC score at the start of treatments, the lower the DIC improvement rate and the higher the deterioration rate. Thus, advanced DIC cases would have poorer outcome, and earlier implementation of treatment is required to improve outcome in patients with DIC.

On the basis of the above reasons, making a diagnosis in the early stage of DIC, followed by implementation of treatments right after the diagnosis, is currently thought to contribute to improvement in outcome. Therefore, it is suggested that treatments should be initiated with early diagnosis by using the JAAM DIC diagnostic criteria. However, other diseases that show similar conditions to DIC should first be excluded.

CQ4: What kind of therapeutic agent should be used for septic DIC?

A4: The currently available therapeutics include unfractionated heparin (UFH) (2D*), low molecular weight heparin (LMWH) (2C*), danaparoid sodium (DS) (2D*), antithrombin III (ATIII) preparation (2C), and recombinant human thrombomodulin (rhTM) (2C*).

Comment: Treatment for causative pathophysiology is given the highest priority even in the treatment of septic DIC. In parallel with this, anticoagulation therapy would also be important. Anticoagulants [264] such as heparin and heparinoids (UFH, LMWH, and DS) themselves do not show an anticoagulant effect but have the potential to improve DIC by enhancing the antithrombin activity of antithrombin. However, the use of heparin is not recommended for patients with bleeding and those with renal or hepatic dysfunction. On the other hand, LMWH and DS, as compared with UFH, are known to have higher anti-factor Xa activity than antithrombin activity [264].

1. UFH
  No RCT has validated the effects of UFH on DIC. Only its inferiority compared with rhTM and other anticoagulants has been reported [265]. Also, the KyberSept study reported that UFH with concomitant high-dose ATIII administration promoted bleeding in patients with sepsis [266]. Currently, UFH may be used for DIC treatment; however, the recommendation level is low. In the presence of the complication of thromboembolism, UFH can be used with particular attention to bleeding.
2. LMWH
  Only dalteparin is approved for DIC in Japan. A multicenter double-blind study on DIC reported that dalteparin reduced the occurrence of organ failure, alleviated bleeding symptoms, and showed higher safety compared with UFH [267].
3. DS
  A multicenter randomized study demonstrated that DS showed no significant differences in both effects and safety in DIC as compared with UFH [268].
4. Antithrombin
  The 2008 SSCG recommended that antithrombin should not be used in the treatment of severe sepsis and septic shock. As an evidence for this statement, the guidelines referred to a large-scale prospective RCT on high-dose antithrombin (KyberSept study) in adult patients with severe sepsis and septic shock, which reported in 2001 that antithrombin did not bring beneficial effects on all-cause 28-day mortality and that concomitant use of heparin increased the risk of bleeding [266]. However, a subgroup analysis in 2006 reported that antithrombin reduced the 90-day mortality in septic patients without a concomitant use of heparin [269]. Furthermore, in severe sepsis patients complicated with DIC, antithrombin was reported to improve the 90-day outcome [270]. In Japan, according to expert consensus opinion in light of the above reports [269],[270], the use of antithrombin alone (without concomitant heparin) is recommended, although weakly, in septic patients complicated with DIC [264]. However, the dose of antithrombin in the KyberSept study is extremely high compared with those used in Japan; therefore, care must be taken in interpreting the results even in subgroup analysis.
5. Thrombomodulin
  Thrombomodulin activates protein C through the generation of the thrombin-thrombomodulin complex and exert the effect of activated protein C (APC) *in vivo*[271]. rhTM is an agent developed as a soluble protein containing extracellular domain that is required for the expression of the function of thrombomodulin. rhTM reversibly binds with thrombin to form a complex that activates protein C. Furthermore, APC in combination with protein S inactivates coagulation factor Va and factor VIIIa, resulting in the suppression of further production of thrombin [265],[271]. In addition to its anticoagulatory effect, thrombomodulin has antifibrolytic effects through the activation of the thrombin-activatable fibrinolysis inhibitor [272]. Furthermore, rhTM is reported to adsorb HMGB-1, neutralize and degrade the adsorbed HMGB-1, and suppress HMGB-1-mediated inflammatory responses through receptor for advanced glycation end products (RAGE). Furthermore, rhTM was reported to bind lipopolysaccharide; thus, rhTM has expedient pharmacologic properties as a therapeutic drug for septic DIC [273]. A multicenter double-blind RCT comparing rhTM and heparin groups was conducted in 234 patients with DIC. As a result, the improvement rate of DIC was 66.1% in the rhTM group and 49.9% in the heparin group. Also, this trial reported that the rhTM group showed improvement in the clinical manifestation of bleeding and DIC as compared with the heparin group [265]. In addition, it has been reported that the 28-day mortality was significantly lower in the rhTM group than in historical controls in mechanically ventilated septic patients complicated with DIC [274].

CQ5: Are protease inhibitors effective for septic DIC?

A5: Synthetic protease inhibitors (SPIs), including gabexate mesilate (GM) and nafamostat mesilate (NM), demonstrated equivalent effects to UFH (2D*); thus, their use may be suggested if active bleeding or hemorrhagic complications are expected (2D*).

Comment: Because SPIs produce effects even in the absence of antithrombin, they may be used even in DIC patients with lowered antithrombin activities. SPIs are approved for DIC by the Japanese health insurance system and cause hemorrhagic complications less frequently than do heparin and heparinoids; thus, they have been used frequently in clinical practice.

1. GM
  Only two RCTs conducted in a single center have been conducted on the use of GM for the treatment of DIC [275],[276]. One is an RCT involving adult patients with DIC, excluding those with hematological and obstetric diseases [275]; the other is an RCT targeting ICU patients who developed DIC from infection after abdominal surgery, in which no significant difference in morality was found between a group treated with GM and a group treated with saline [276].
  An unblinded multicenter RCT in Japan [277] involved patients in whom DIC was diagnosed by using the researchers’ own criteria. In this study, 211 patients were enrolled and 203 patients (109 in the GM group, 94 patients in the UFH group) were analyzed [277]. The overall survival rate was not significantly different between groups. The number of deaths attributable to DIC was significantly lower in the GM group than in the UFH group (10 of 109 in the GM group and 19 of 94 in the UFH group) (*p* =0.028). There was no difference in the improvement of bleeding symptom between groups; however, the UFH group had significantly more patients whose conditions deteriorated (*p* <0.01).
2. NM
  A multicenter RCT was conducted in the research and development phase of NM in 57 hospitals in Japan [278]. This was an unblinded RCT in 163 patients (82 in the NM group and 81 in the UFH group) with DIC or suspected DIC according to the DIC criteria of the MHLW, in which 0.2 mg kg^−1^ h^−1^ NM or 10 IU kg^−1^ h^−1^ UFH was administered, respectively. The NM group showed significant improvement in organ manifestations (primary physician’s judgment at the final day, *p* <0.05) and antithrombin activity (*p* <0.01) compared with the UFH group; however, no difference was found in the DIC score. Most of the cases enrolled in this study were leukemia and malignant tumors, and there were only six cases of DIC resulting from infections.
  A search of the literature showed that these two SPIs have possible effects equivalent to those of UFH, including improvement in prognosis, complication, and coagulation variables. However, no RCT on UFH targeting septic DIC exists; therefore, the recommendation for the use of SPIs is considered to be limited.

CQ6: Is blood transfusion done for septic DIC?

A6: Blood transfusion is usually not recommended. However, it can be used in combination with anticoagulant administration if a bleeding tendency is apparent owing to a decrease in each blood component (1D*).

Comment:

1. Fresh frozen plasma (FFP)
  In patients showing a marked bleeding tendency, when the activated partial thromboplastin time or prothrombin time-international normalized ratio is increased to a level more than double of the normal, FFP administration is indicated.
2. Platelet concentrates (PC)
  If a marked tendency for bleeding is observed, the platelet count is <50,000/mm^3^, and surgery or vessel puncture is required, PC should be administered carefully. Especially in cases of septic DIC, if the patient does not receive appropriate anticoagulant therapy including rhTM or ATIII preparations, further deterioration of organ functions might occur. Furthermore, PC is contraindicated for heparin-induced thrombocytopenia, and if ADAMTS-13 is markedly reduced to ≤3% in thrombocytopenic thrombotic purpura, PC transfusions should be very carefully performed [264].

### Acute blood purification

CQ1: When should renal replacement therapy (RRT) for septic acute kidney injury (AKI) be initiated?

A1:

- There are no explicit criteria for the timing of the initiation of RRT on the basis of renal function indices, including blood urea nitrogen (BUN) and creatinine (Cre) (2C*).
- However, early initiation of RRT is recommended in severe sepsis and septic shock if the urine output is not restored after adequate fluid resuscitation (1C*).

Comment: Two RCTs with level B or higher evidence [279],[280] and three prospective observational studies [281]-[283] reported since 2000 were reviewed. No study designed for and focused on sepsis patients was found. No evidence was found for using the value of BUN, Cre, or urine volume for the timing of the initiation of RRT. Bagshaw et al. [282] reported in an international multicenter observational study (BEST kidney study) in 2009 that early RRT initiation for AKI significantly reduced mortality: 58.9% mortality for RRT initiation within 2 days after admission; 62.1%, 2–5 days; and 72.8%, >5 days. Similarly, Payen et al. [283] reported from the analysis of the SOAP study that although patients in the early RRT group (within 2 days) had higher severity on ICU admission, both the 60-day mortality and ICU mortality were significantly lower in the early RRT group. Additionally, patients in the early RRT group showed a tendency to have an even higher serum Cre level (*p* =0.06).

Seabra et al. in 2008 [284] and Karvellas et al. in 2011 [285] reported in their meta-analyses that early RRT initiation for acute renal failure failed to show improved survival rate, and therefore, the usefulness of early RRT initiation remains uncertain. However, these evaluations were mainly for acute renal failure and not for sepsis, and BUN and Cre were used as criteria for the initiation of RRT. In particular, sepsis with acute renal failure is accompanied by systemic inflammation, and therefore, early RRT initiation may be considered before extreme metabolic abnormalities or life-threatening complications progress. In fact, 48% of the patients (594 of 1,238) included in the report by Bagshaw et al. had acute renal failure caused by septic shock. Also, the RENAL study reported in the *New England Journal of Medicine* in 2009 showed better results (a survival rate of 55.3% and renal recovery rate of 94% among survivors) than previous studies [286]. These results were attributed to the use of continuous renal replacement therapy (CRRT) as an initial treatment in all patients and to the initiation of RRT within 50 h after ICU admission, which was earlier than in previous studies [286]. In this regard, early initiation of CRRT is suggested to contribute to better survival and recovery rate of renal function [287].

CQ2: Which modality of RRT for septic AKI should be used, CRRT or intermittent renal replacement therapy (IRRT)?

A2:

- There is no evidence suggesting that CRRT improves outcome better than IRRT (2A*).
- However, CRRT or sustained low-efficiency dialysis (SLED) is recommended rather than IRRT for hemodynamically unstable patients, considering the management of fluid balance (1C*).

Comment: Two RCTs with level A evidence [288],[289], five RCTs with level B evidence [290]-[294], and two observational studies with level B evidence [295],[296] reported since 2000 were extracted. Among these articles, the report by John et al. [294] was targeted on sepsis. Among these seven RCTs, the report by Mehta et al. [290] showed that mortality was significantly higher with CRRT than with IRRT, whereas the other six reports showed no difference in mortality between CRRT and IRRT. Among these six reports, the Hemodiafe study (359 patients at 21 centers in France) reported in the *Lancet* by Vinsonneau et al. in 2006 [288] showed that there was no difference in the 28-day, 60-day, and 90-day survival rate and the dialysis dependence rate. However, it was pointed out in the *Lancet* that the use of a highly biocompatible dialyzer in the IRRT group affected the results in this study [297]. Lins et al. [289], in the SHARF study (316 patients at nine centers in Belgium) in 2009, reported that in-hospital mortality, renal recovery rate, and the duration of ICU stay did not differ between two groups. However, hemodynamically unstable patients were excluded in this study. The results of meta-analyses by Bagshaw et al. [298] and Pannu et al. [299] showed no difference in prognosis between CRRT and IRRT. As stated above, no evidence has been obtained showing that CRRT improves prognosis better than IRRT.

However, CRRT is recommended over IRRT for hemodynamically unstable patients in the 2004 and 2008 SSCG. In fact, the protocol of the Acute Renal Failure Trail Network (ATN) study, discussed below, was designed to select CRRT as the modality for hemodynamically unstable patients. Many published reports described that fluid overload is an important factor associated with increased mortality in critically ill patients, and CRRT, which provides flexibility and control in fluid management, is considered to be useful in the management of critically ill patients [300]. In published reports, SLED is comparable to CRRT with regard to the influence on hemodynamics; therefore, it is an alternative modality for hemodynamically unstable patients [301]. In Japan, continuous hemodiafiltration (CHDF) is the most popular mode of RRT for acute renal failure [302]. On the other hand, the first investigation by the Sepsis Registry Committee of the JSICM [44] reported that continuous hemo(dia)filtration or intermittent dialysis (IHD) was performed in 104 of 266 patients: CHDF in 68% (71 of 104 patients), continuous hemodialysis (CHD) in 7% (7 patients), continuous hemofiltration (CHF) in 5% (5 patients), IHD in 10% (10 patients), and other high-efficiency blood purification such as high flow volume HDF in 11% (11 patients); 80% of these treatments were performed as continuous therapy. Significantly higher APACHE II score (23.3) and 28-day mortality (54.8%) were observed in the blood purification group compared with the non-blood purification group.

CQ3: What is the optimal dose in RRT for septic AKI?

A3: Although several RCTs with high level of evidence studying the relation between the dose of blood purification (sum of the dialysate flow volume and filtrate volume) and prognosis exist, the optimal dose remains unclear (1A*).

Comment: Six RCTs with level A evidence [286],[303]-[307], three RCTs with level B evidence [279],[308],[309], and four meta-analyses [310]-[313] reported since 2000 were extracted. Among these, the report by Zhang et al. [307] was targeted on sepsis. In 2000, in an RCT reported in the *Lancet*, Ronco et al. [303] concluded that increasing the filtrate volume in CHF was useful because the 15-day survival rate was significantly higher in the 35 and 45 mL kg^−1^ h^−1^ filtrate rate groups than in the 20 mL kg^−1^ h^−1^ group, and subanalyses in patients with sepsis showed a significant improvement of survival rate in the 45 mL kg^−1^ h^−1^ group. In 2006, Saudan et al. [304] reported that the 28-day survival rate was significantly higher in the CHDF group with an additional dialysate flow rate of 18 mL kg^−1^ h^−1^ than in the CHF group with a filtrate volume of 25 mL kg^−1^ h^−1^, thus demonstrating that an increase in dose was useful. On the other hand, Tolwani et al. [305] reported in 2008 that there was no significant difference in the 30-day survival rate, ICU survival rate, and in-hospital survival rate between CHDF with a total dose of 20 mL kg^−1^ h^−1^ and that with 35 mL kg^−1^ h^−1^. These results warranted a large-scale RCT; hence, the ATN and RENAL studies were planned. In 2008, the ATN study (1,124 patients with AKI) reported in the *New England Journal of Medicine* proved that there was no added benefit from an intensive treatment strategy (IHD six times weekly or CRRT of 35 mL kg^−1^ h^−1^) as compared with a less intensive strategy (IHD three times weekly or CRRT of 20 mL kg^−1^ h^−1^), resulting in 53.6% and 51.5% 90-day mortality rate, respectively [306]. In 2009, the RENAL study (1,464 patients with AKI) reported in the *New England Journal of Medicine* demonstrated that treatment of higher intensity (40 mL kg^−1^ h^−1^) did not reduce the mortality at 90 days compared with the lower-intensity treatment (25 mL kg^−1^ h^−1^), resulting in a 90-day mortality of 44.7% in both groups [286]. Following these results, a joint statement by five societies, including the ATS, on the prevention and management of acute renal failure in ICU patients [314] recommends an actual delivered dose of at least 20 mL kg^−1^ h^−1^, whereas no evidence for recommending the dose of 20 mL kg^−1^ h^−1^ or recommending against the dose of ≥40 mL kg^−1^ h^−1^ was found.

CQ4: Is (continuous) hemo(dia)filtration effective for severe sepsis?

A4:

- Selection of a membrane with adsorption property or a membrane with a large pore size, or an increase of dose, is required to eliminate mediators including cytokines (2C).
- With the above methods, there is a possibility to improve hemodynamics (2C).
- However, there is no evidence showing that those methods improve outcome (2C).

Comment: Seven RCTs with level B evidence or higher and one cross-over trial report since 2000 were extracted. Hemo(dia)filtration techniques, including increased dose, use of a membrane with adsorption property, or use of a membrane with a large pore size, have been attempted for severe sepsis in the clinical setting. Concerning the attempt to increase the dose, there were three reports on septic shock [315]-[317] comparing a high filtration rate (4–6 L/h) and a normal filtration rate (1–2 L/h). Significantly improved hemodynamics, decreased noradrenaline [316],[317], and reduced blood cytokine level in the high filtration rate group were reported [315],[317].

Furthermore, as a method based on the principle of adsorption, a total of four RCTs [318]-[321] compared a CH(D)F group with use of the AN69 hemofilter, which has high absorptive capacity for cytokines, and a conservative therapy group for sepsis. Three [319]-[321] of the four RCTs demonstrated significantly decreased blood cytokine level with CHDF with the AN69 hemofilter. In 2010, Peng et al. [319] showed significantly reduced blood levels of not only inflammatory cytokines but also anti-inflammatory cytokines, as well as significantly increased monocytic HLA-DR expression rate, in a group treated with CHF with the AN69 hemofilter. On the other hand, Payen et al. [322] performed an RCT comparing a conservative therapy group and a group treated with CHF for 96 h by using the PS hemofilter instead of AN69 in early-stage patients with severe sepsis. As a result, early CHF initiation caused deteriorated outcomes and prolonged use of organ supports, including mechanical ventilator and catecholamine administration. In contrast, de Pont [323] pointed out in an editorial in the same journal that the filtration rate was low at 2 L/h, and the AN69 hemofilter should have been used because cytokines are deeply involved in the pathophysiology of sepsis. Furthermore, high-cutoff hemofilters having larger pore sizes are in clinical use for cytokine removal in foreign countries [324].

However, the abovementioned statement by the ATS and other societies recommended against high-flow hemofiltration for severe sepsis or septic shock without renal failure [314]. In Japan, there is an upper limit for the volume of replacement fluid in health insurance, and therefore, the use of cytokine-absorbing hemofilter (PMMA membrane hemofilter) is practical, as Oda et al. advocated [325], and the strategy of using the principle of adsorption is advocated in foreign studies [326]. In Japan, a clinical trial on the AN69ST hemofilter, which is made of modified AN69 membranes, has just been completed. In the previously mentioned RENAL study, all patients were treated by using AN69 (including AN69ST) hemofilters; this study showed higher survival rates than previous studies with a similar degree of patient severity. However, the evidence is insufficient to conclude whether prognosis is improved. As an additional description on the current situation in Japan, although CH(D)F is not approved by health insurance for the treatment of severe sepsis itself, these methods were initiated in 66 of 100 patients (66%) for renal indications and 34 patients (34%) for nonrenal indications according to the abovementioned investigation by the Sepsis Registry Committee [44].

CQ5: Is polymyxin B-immobilized direct fiber hemoperfusion (PMX-DHP) effective for septic shock?

A5:

- Improvements in hemodynamics and respiratory function were demonstrated in septic shock requiring emergency abdominal surgery (2C).
- The evidence is insufficient to conclude whether prognosis is improved (2C).

Comment: Two RCTs with level B evidence reported since 2000 were extracted [327],[328]. A pilot controlled study, which was performed at six ICUs in Europe, reported in 2005 showed no significant difference in the survival rate, blood endotoxin level, blood IL-6 level, and improvement of SOFA score between the group treated with PMX-DHP (direct hemoperfusion using polymyxin B-immobilized endotoxin removal cartridge) and patients with septic shock due to postoperative or intra-abdominal infections who received standard treatment [327]. However, the cardiac index, left ventricular stroke work index, and oxygen delivery index were significantly improved. A systematic review reported in 2007 compared a PMX-DHP treatment group (978 patients) and a conventional treatment group (447 patients) for infections including those other than abdominal infections or non-gram-negative bacterial infections and found improvements in elevated mean arterial pressure, reduced dose of catecholamine, and elevated PaO_2_/F_I_O_2_ ratio [329]. However, the results were unreliable because the 28 articles were mostly case reports from Japan and overlapping data from the same center were included.

The Early Use of Polymyxin B Hemoperfusion in Abdominal Sepsis (EUPHAS) trial published in *JAMA* in 2009 demonstrated significant improvement in respiratory function, SOFA score, and 28-day mortality, in addition to hemodynamic improvement by PMX-DHP in patients with severe sepsis and septic shock due to intra-abdominal infections that required emergency surgery. The trial was terminated after enrolling 64 patients because the improvement in the 28-day mortality met the criteria for trial termination through interim analysis [328]. However, Vincent, in a letter to the editor [330], pointed out that no statistically significant difference was found in the survival rate and that there were other concerns on the distribution of pathogenic bacteria between groups. Thus, it is still unclear whether PMX-DHP improves prognosis. There is a report from Japan showing that the hospital mortality of patients with lower intestinal perforation requiring emergency surgery, which is almost the same indication as in the EUPHAS trial, was 17.9% without PMX-DHP treatment, which was lower than the predicted mortality rate of 63.3% based on APACHE II scores [331]. The main mechanism of action of PMX-DHP is adsorption of endotoxin, although effective mechanisms other than endotoxin adsorption are reported [332]. On the basis of the understanding of the pathophysiology of sepsis, endotoxin does not play a key role but rather functions only as one of the pathogen-associated molecular patterns. Therefore, there are doubts about the efficacy against sepsis of a blood purification method that removes only endotoxin [333].

The abovementioned first investigation of the Sepsis Registry Committee [44] showed that PMX-DHP was performed in 43 of 266 patients (16%). The group treated with PMX-DHP showed both significantly higher APACHE II score (23.1) and 28-day mortality (44.2%) compared with the group without PMX-DHP. Additionally, the cases were matched by using propensity score analysis because the severity was different between the groups. The covariates included for propensity matching were four background items (age, sex, APACHE II, and SOFA score) and eight items of laboratory data before PMX treatment or interventions (lactate measurement, antibiotic administration within 1 h after admission, implementation of EGDT, etc.). Thirty-seven matched cases from each group were examined for prognosis. As a result, the 28-day mortality rate of 37 patients treated with PMX-DHP (37.8%) was significantly lower than that of 37 patients without PMX-DHP (67.6%) (*p* =0.019). In addition, the rate of implementation of intensive insulin therapy was significantly higher in the PMX-DHP group, among the items of sepsis bundle treatment after the implementation of PMX treatment.

### Abbreviations

RRT renal replacement therapy

CRRT continuous renal replacement therapy

IRRT intermittent renal replacement therapy

IHD intermittent hemodialysis

HDF hemodiafiltration

CHF continuous hemofiltration

CHD continuous hemodialysis

CHDF continuous hemodiafiltration

SLED sustained low-efficiency dialysis

AKI acute kidney injury

PMMA polymethylmethacrylate

PMX-DHP polymyxin B-immobilized direct fiber hemoperfusion

PS polysulfone

### Immunoglobulin

CQ1: What is the indication for immunoglobulin administration in septic patients?

A1: Currently, there is insufficient evidence suggesting that immunoglobulin administration improves the prognosis of adult patients with sepsis (2B). However, with a reduced duration of mechanical ventilation and improvement in ICU survival, administration of immunoglobulin may be considered (2C).

Comment: Immunoglobulin preparations contain specific antibodies against various bacteria, toxins, and viruses. When combined with an antigen, immunoglobulin exerts an opsonic effect and complement-activating effect, neutralizes activity against toxins and viruses, suppresses pro-inflammatory cytokines, has an antibody-dependent bactericidal activity-enhancing function, and further increases the susceptibility to antimicrobial agents by directly acting on the cell wall of pathogenic microorganisms [334],[335]. Consequently, immunoglobulin is used as an adjuvant therapy for infections. The blood levels of gamma globulin in the early stage of septic shock were reported to be reduced to abnormally low levels owing to depressed production, leakage, or consumption [336]. The incidence of shock and mortality were significantly high in patients with sepsis with low gamma globulin level [337]; however, mortality was improved if the initial antimicrobial administration was appropriate and immunoglobulin was additionally administered [338]. On the other hand, if the initial antimicrobial therapy was inappropriate, the prognosis was not improved with immunoglobulin administration alone [339].

Four RCTs [339]-[342] were reported since 2000 on immunoglobulin administration against sepsis or septic shock. Among them, two reports were large-scale RCTs with >100 patients in each group [339],[342]. The study by Masaoka et al. [339], which was an unblinded RCT in patients with refractory infections complicated with mainly blood diseases, reported defervescence effects and prognosis improvement by immunoglobulin administration (5 g for 3 days). On the other hand, Werdan et al. reported in 2007 [342] that significant improvement in APACHE II score, ICU survival, and reduction of the duration on mechanical ventilation were observed with immunoglobulin administration (for 2 days: day 1, 0.6 g/kg; day 2, 0.3 g/kg), although the 28-day mortality was not significantly improved. The difference between the two studies may be attributed to the difference of severity (higher severity in Werdan et al.’s study: 75% patients complicated with shock and mean APACHE II score of 28) and to the normal IgG levels before immunoglobulin administration.

Following the previously published five meta-analyses [343]-[347], Alejandria et al. [348] conducted a meta-analysis in 2010 on 42 RCTs (adult sepsis in ten articles) comparing an immunoglobulin administration group and a control group (placebo or nonadministration group) in patients with bacterial sepsis or septic shock, on the basis of three databases (Cochrane Review, MEDLINE, and EMBASE). As a result, a significant reduction in the 30-day mortality in the immunoglobulin administration group was found. However, there was no significant difference in mortality when the analysis was limited to articles with a low risk of bias.

However, as the study by Werdan et al. (published in 2007) [342] was performed in 1991–1995 (i.e., before the publication of the SSCG in 2004), there are many differences in therapies, including early administration of antimicrobials or implementation of EGDT, in addition to the differences in the definition of sepsis and severity score, from the contents of the SSCG being implemented currently.

The 246 patients with sepsis enrolled in the first investigation by the Sepsis Registry Committee of the JSICM in 2007 [44] were classified into two groups according to the administration of immunoglobulin, and the outcome was compared by using propensity score analysis in 70 patients in each group (mean APACHE II score: 19–20, SOFA score: 8) matched by four background factors (age, sex, APACHE II, SOFA score) and seven factors of laboratory data before treatments (blood lactate, blood culture before administration of antibiotics, antibiotic administration within 1 h after ICU admission, etc.). Although the dose of immunoglobulin administered (maximum of 15 g for 3 days) was smaller than in previous reports, significant improvements in the 28-day, ICU, and in-hospital mortality were found in the immunoglobulin administration group. In addition, among the treatments recommended in the sepsis bundle, the rate of steroid administration was significantly higher in the immunoglobulin administration group.

Immunoglobulin therapy is an adjunctive therapy for the treatment of infection. Therefore, immunoglobulin may exert its effects through the treatments recommended in the SSCG, such as EGDT to improve tissue oxygen metabolism or thorough performance of blood culture, and early administration of antibiotics, which are the mainstays of sepsis treatment. A large-scale RCT to evaluate the effect of immunoglobulin administration is required in the future.

CQ2: When should immunoglobulin be administered?

A2: Immunoglobulin administration may be considered in the early stage of sepsis (2C).

Comment: There is no RCT on the timing of the initiation of immunoglobulin administration in patients with sepsis. However, Berlot et al. [349] retrospectively studied the prognosis and initiation time of immunoglobulin administration in 129 patients with severe sepsis/septic shock and found that the surviving group received significantly earlier administration than the deceased group (23 h vs. 63 h). Turgeon et al. [345] performed a meta-analysis of 20 RCTs on immunoglobulin administration in adult patients with sepsis in 2007 and reported a significant improvement in 30-day mortality. In this report, the timing of immunoglobulin administration was on the day of sepsis diagnosis in 18 reports, on the next day after the diagnosis in 1 report, and on the third day after the diagnosis in the remaining 1 report. “Initiation of administration on day 3 of onset of sepsis” was indicated in the report by Masaoka et al. [339] in patients with refractory severe infection who had no response to 3 days of antimicrobial administration. With respect to the initiation time of administration, both the group that was initiated on the same day as the diagnosis (18 articles: relative risk (RR), 0.75) and the group initiated on day 3 after the diagnosis (20 articles: RR, 0.74) showed a significant improvement in 30-day mortality. Accordingly, immunoglobulin should be administered in the early stage of onset.

CQ3: What should be the dose and duration of immunoglobulin administration?

A3: A total immunoglobulin dose of ≥0.2 g/kg should be administered for ≥3 days (2C).

Comment: There is no RCT on the dose of immunoglobulin (i.e., whether or not there is a dose dependency of the effect) in patients with sepsis. In 2007, Turgeon et al. [345] performed a meta-analysis of 20 RCTs on immunoglobulin administration in adult patients with sepsis and investigated the total dose of immunoglobulin and the duration of administration. The total dose was 0.2–1.75 g/kg (conversion based on a body weight of 75 kg, mean 0.90 ± 0.46 g/kg), and the duration of administration was 2–5 days (mean 3.0 ± 0.97 days). A study of 30-day mortality was performed by classifying patients into groups that received ≥1 g/kg and <1 g/kg of total dose. The results showed that reduced mortality was found in both groups and that the group treated with ≥1 g/kg had a significantly smaller RR. A study of 30-day mortality was performed by classifying patients into a group with ≥3 days and a group with ≤2 days of administration. Reduced mortality was found only in the group with ≥3 days of administration.

Under the health insurance system of Japan, the indications for immunoglobulin therapy for infection are severe infections, viral infections, and agamma- or hypogamma-globulinemia, but not sepsis. Usually, the indicated dose is 5 g daily for 3 days (based on a body weight of 75 kg: 15 g/75 kg =0.2 g/kg). This dose corresponds to the dose that Masaoka et al. [339] used in a multicenter unblinded RCT for the reevaluation of immunoglobulin, under the direction of the MHLW.

The 246 patients with sepsis enrolled in the first investigation by the Sepsis Registry Committee of the JSICM in 2007 [44] were classified into two groups according to the administration of immunoglobulin, and the outcome was compared by using propensity score analysis in 70 patients in each group (mean APACHE II score: 19–20, SOFA score: 8) matched by four background factors (age, sex, APACHE II, SOFA score) and seven factors of laboratory data before treatments (blood lactate, blood culture before administration of antibiotics, antibiotics administration within 1 h after ICU admission, etc.). Although the dose of immunoglobulin administered (maximum of 15 g for 3 days) was smaller than in the studies reported in Europe and the United States, significant improvements in the 28-day, ICU, and in-hospital mortality were found in the immunoglobulin administration group [44]. In addition, among the treatments recommended in the sepsis bundle, the rate of steroid administration was significantly higher in the immunoglobulin administration group.

From the above discussion, a total immunoglobulin dose of ≥0.2 g/kg, or if possible ≥1 g/kg, for ≥3 days is recommended.

CQ4: What should be given particular attention in the selection of immunoglobulin preparation?

A4: Use of a complete-molecular-type preparation is suggested (2C).

Comment: An immunoglobulin preparation with incomplete molecular type lacks Fc gamma receptor, and thus has no opsonic effect, and has a reduced half-life in blood. Therefore, a complete-molecular-type immunoglobulin preparation should be used, in which the normal antibody structure is not destroyed and Fc gamma receptor function is maintained. IgM-enriched polyclonal immunoglobulin has received attention overseas because it was reported to improve the 30-day mortality; however, a recent RCT failed to show its effect [341].

Generally, the frequency of adverse effects of immunoglobulin is 5%–10%; anaphylaxis tends to develop especially in patients with IgA deficiency or anti-IgA antibody [350]. If a blood preparation containing IgA is administered to a patient with IgA deficiency, anti-IgA antibody is produced and causes fatal anaphylactic shock. The rate of a serum IgA level of ≤5 mg/dL in Japanese patients is 0.03%–0.05%. The onset of adverse effects often occurs within 1 h after the start of infusion. Skin reactions (allergy, redness, rash, pruritus), renal dysfunction, aseptic meningitis, anaphylaxis, thromboembolism, viral infection, and others are reported as adverse drug reactions [343],[350]; however, serious adverse effects are very rare, and consequently, cases of death are very few.

### Protease inhibitor

CQ1: What is the indication for synthetic protease inhibitors (SPIs) in sepsis?

A1:

- Ulinastatin (UTI): evidence on its efficacy for septic shock is insufficient (2D).
- Sivelestat sodium (sivelestat): may be considered in patients with ALI/ARDS (2C*).

Comment: SPIs are not approved for sepsis itself by health insurance; however, many literature reports have shown the effects of SPIs on various pathophysiologies, such as circulatory failure, respiratory failure, and DIC due to sepsis. Sepsis-related articles were collected. Although the literature review in this guideline focused on articles published since 2000 for foreign literature and since 1991 for Japanese literature, articles concerning SPIs were searched dating back to earlier, based on the historical background that these medications were developed and had been used for a long time only in Japan.

The indications for UTI and sivelestat for sepsis are discussed below.

1. UTI
  The indications for UTI are acute circulatory failure (bacterial, hemorrhagic, traumatic, and burn) and acute pancreatitis; this agent has been used for a long time. In a double-blind study in 40 institutions targeting patients with shock excluding those with cardiogenic shock, a significant improvement in shock score evaluated with blood pressure, heart rate, base excess, urine output, and level of consciousness was reported. UTI was more effective in the high-dose group than in the low-dose group, and a significant improvement of prognosis in septic shock was achieved [351]. A double-blind study performed in six institutions [352] suggested the possibility that UTI improves hemodynamics through suppressing the release and inhibiting the activity of proteases, because UTI itself has no direct effect on hemodynamics. Furthermore, UTI has also been found to have a free radical scavenging activity [353]. In RCTs targeting sepsis performed in China, improvements in the severity score, homeostasis, and survival have been reported [354]-[357]. However, the method of administration was different from that in Japan with regard to the dose of UTI and the combination use of thymosin α.
2. Sivelestat
  The indication for sivelestat, a neutrophil elastase inhibitor, is acute lung injury associated with SIRS. Many patients with sepsis are complicated with ALI/ARDS, and one of the causes of ALI is lung injury caused by elastase released from neutrophils sequestered to the lung.
  The STRIVE study, a multicenter RCT performed abroad, failed to show the usefulness of sivelestat although the mortality rate was slightly increased [358]. Other than the improvement in mortality, sivelestat was also reported to improve the DIC score and PaO_2_/F_I_O_2_ ratio owing to the suppression of pulmonary capillary permeability. Furthermore, the length of ICU stay was shortened [359]-[361].
  It is expected that inflammation develops early in severe ARDS. Thus, sivelestat is reported to be effective if administered in the early stage from the onset of lung injury [362].
  In a multicenter study reported in 2011, which was performed in Japan and enrolled 581 cases, improvement of the rate of weaning from mechanical ventilation, reduction of ICU stay, and significant improvement of survival rate were observed [363]. Therefore, the possibility that the effect was diminished by the delay in the timing of administration in the STRIVE study cannot be denied. Further study would be necessary in the future.

## Summary

The draft of this guideline was first presented in March 1, 2012, at the 39th Annual Meeting of the JSICM and was open to the public through the website of the JSICM from May 1. After 1 month of recruitment of public comments, the guideline was revised and finalized by the Sepsis Registry committee on August 11, 2012.

These guidelines focused on unique therapies in Japan that were overlooked in many Western guidelines and on items for which differences in opinion exist between Japan and Western countries, rather than covering all items related to diagnoses and therapies of sepsis like the SSCG do. The SSCG are evidence based and are the world’s first clinical practice guidelines for sepsis. Moreover, the SSCG make excellent points in the introduction of the timing concept for sepsis therapy and in efforts to standardize various therapies based on evidence, and hence we have no intention to deny the importance of the utility of SSCG. On the other hand, the health insurance system in Japan is different from Western health insurance systems, and sepsis treatment results in Japan are never inferior to those in Western countries. Japan’s unique therapies are also performed on the basis of evidence. We made efforts to make these guidelines easy to understand and use in clinical practice, and provide concrete information according to the opinions of the members of the JSICM.

Although these guidelines describe the standard clinical practice for sepsis in Japan, we do not intend to force these recommendations. The best therapy should be based on the physician’s judgment according to the conditions of individual patients in actual clinical settings.

Lastly, we would like to deeply acknowledge all institutions that cooperated in the two investigations conducted by the Sepsis Registry Committee of the JSICM and to all persons concerned with the creation of these guidelines.

### Disclosure of conflict of interest (COI)

Dr. Oda received lecture fees from MSD Co. Ltd., and his institution received scholarship donation from Torii Pharmaceutical Co., Ltd. Dr. Endo consulted for Kyowa Hakko Kirin Co., Ltd. Dr. Noguchi holds stocks of YUFU Research Co., Ltd. Dr. Matsuda received lecture fees from Asahi Kasei Pharma Corporation, Ono Pharmaceutical Co, Ltd., Pfizer Japan Inc., and Taisho Toyama Pharmaceutical Co., Ltd. He also received manuscript fees from Gakken Medical Shujunsha Co., Ltd., and his institution received research funds from Dainippon Sumitomo Pharma Co., Ltd. Other authors have disclosed that they have no potential conflicts of interests.

## Additional file

## Electronic supplementary material

Additional file 1:**Supplementary file.** This file contains tables on the evidence level of the literature, quality of recommendations, strength of recommendation, factors determining the strength of recommendation, supplemental signs and variables for the diagnosis of sepsis, recommended empirical antibiotic regimens, definitive antibiotic therapy, treatment option for highly drug-resistant and/or multidrug-resistant organisms, PD parameters of representative classes of antibiotics, and standard duration of antibiotic therapy for representative infectious diseases, and a figure showing an example of initial resuscitation. (DOCX 98 KB)
