# Supplementary material for: The Japanese guidelines for the management of sepsis
Source: J Intensive Care. 2014 Oct 28;2:55. doi: 10.1186/s40560-014-0055-2 (PMC4336273; doi:10.1186/s40560-014-0055-2)
Supplement: Supplementary file 1 — Additional file 1:Supplementary file. This file contains tables on the evidence level of the literature, quality of recommendations, strength of recommendation, factors determining the strength of recommendation, supplemental signs and variables for the diagnosis of sepsis, recommended empirical antibiotic regimens, definitive antibiotic therapy, treatment option for highly drug-resistant and/or multidrug-resistant organisms, PD parameters of representative classes of antibiotics, and standard duration of antibiotic therapy for representative infectious diseases, and a figure showing an example of initial resuscitation. (DOCX 98 KB) [file 40560_2014_55_MOESM1_ESM.docx]

**Table II-1. Ranking of the literature**

| Level of evidence | Study method |
| --- | --- |
| Level A | RCT (randomized controlled trial) |
| Level B | Low-quality RCT or high-quality observational study and cohort study |
| Level C | Observational study with comparison with a control group and cohort study |
| Level D | Case series study or opinion of a specialist |

**Table II-2. Quality of recommendations**

| GRADE A | High-quality evidence: multiple level A study |
| --- | --- |
| GRADE B | Moderate-quality evidence: one level A study |
| GRADE C | Low-quality evidence: level B study only |
| GRADE D | Very-low-quality evidence: level C or lower study |

**Table II-3. Strength of recommendation**

| Recommendation 1 (strong recommendation) | Desired effects (outcome, burden, and cost) according to recommendation apparently exceeding the disadvantage |
| --- | --- |
| Recommendation 2 (weak recommendation) | Although it is anticipated that the desired effects according to the recommendation will exceed the disadvantage, there is a lack of sufficient rationale or paucity of reliability |

**Table II-4. Factors determining strength of recommendation**

| Factors for consideration | Recommendation process |
| --- | --- |
| Quality of evidence | If the quality of evidence is low, a strong recommendation is not given. |
| Relative importance of the outcomes | If the values or criteria vary, a strong recommendation is not given |
| Baseline risks of outcomes | The higher the risk, the greater the benefit, the stronger the recommendation |
| Magnitude of relative risk (including benefit, harm, and burden) | Larger reduction of relative risk makes a strong recommendation; increase in harmful relative risk makes a weak recommendation |
| Absolute magnitude of the effect | If the absolute effect is greater than the harm, a strong recommendation is given; in the opposite case, a weak recommendation is given. |
| Accuracy of prediction of effect | High accuracy: strong recommendation |
| Cost | High cost: weak recommendation |

**Table III-1-1．Supplemental signs and variables for the diagnosis of sepsis**

| General condition variables |  |
| --- | --- |
| Fever | Core temperature >38℃ |
| Hypothermia | Core temperature <36℃ |
| Heart rate | >90/min，or >2 SD above the reference value for age |
| Tachypnea (>20/min) |  |
| Altered mental status |  |
| Significant edema or positive fluid balance (>20 mL/kg in 24 h) |  |
| Hyperglycemia | Blood glucose >120 mg/dL in the absence of diabetes |
| Inflammatory variables |  |
| Leukocytosis | WBC >12,000/μL |
| Leukopenia | WBC <4000/μL |
| With normal white blood cell count, immature white blood cell >10% |  |
| CRP | >2.0 mg/dL* |
| Procalcitonin | >0.5 ng/mL for sepsis, 2.0 ng/mL for severe sepsis |
| IL-6 | >1000 pg/mL* for severe sepsis |
| Hemodynamic variables |  |
| Hypotension | In adults, systolic blood pressure <90 mmHg or mean blood pressure <70 mmHg，or SBP decline >40 mmHg; in children, a decline of >2 SD from the reference value for age |
| Organ dysfunction variables |  |
| Hypoxemia | PaO_2_/F_I_O_2_ <300 |
| Acute oliguria | Urine output <0.5 mL⋅kg^-1^⋅h^-1^ |
| Increase in Cre | >0.5 mg/dL |
| Coagulation disorder | PT-INR >1.5 or aPTT >60 s |
| Ileus | Loss of intestinal peristaltic sound |
| Thrombocytopenia | <100,000/μL |
| Hyperbilirubinemia | T-Bil >4 mg/dL |
| Tissue perfusion variables |  |
| Hyperlactatemia | >2 mmol/L |
| Prolonged capillary refilling time or mottled skin |  |

*Reference value might differ according to the measurement method. aPTT, activated partial thromboplastin time; Cre, creatinine; IL, interleukin; PCT, procalcitonin; PT-INR, prothrombin time-international normalized ratio; SD, standard deviation; T-Bil, total bilirubin.

**Table III-3-１. Recommended empirical antibiotic regimens according to the suspected source of sepsis^#1, 2, 3)^**

| Likely cause of sepsis | Microbiological differential diagnosis | Antibiotic regimens | Note |
| --- | --- | --- | --- |
| Community-acquired pneumonia | - No risk of *Pseudomonas aeruginosa*^#4)^   *Streptococcus pneumoniae*, *Haemophilus influenzae*, *Legionella pneumophila*, *Mycoplasma pneumoniae*   - Risk of *P. aeruginosa*^#4)^   In addition to all of the above  Nosocomial GNB such as *P. aeruginosa* | - No risk of *P. aeruginosa*^#4)^   CTRX (CTX) or SBT/ABPC  plus  AZM  If community-associated MRSA (CA-MRSA) is of concern, add VCM plus CLDM or LZD   - Risk of *P. aeruginosa*^#4)^   CFPM，TAZ/PIPC，or MEPM (DRPM, IPM/CS) ^#5)^  plus  AZM  If CA-MRSA is of concern, add VCM plus CLDM or LZD | Patients with the following risk factors should be treated as having health care–associated pneumonia: antibiotic therapy in the past 3 months, hospitalization $\geq$2 days in the past 3 months, and chronic hemodialysis. |
| Ventilator-associated pneumonia, hospital-acquired pneumonia, nursing and health care–associated pneumonia | - No risk of *P. aeruginosa*^#6)^   *S. pneumoniae*, *H. influenzae*, MSSA, susceptible *Escherichia coli* and *Klebsiella pneumoniae*   - Risk of *P. aeruginosa*^#6)^   In addition to all of the above  Nosocomial GNB such as *P. aeruginosa* and MRSA | - No risk of *P. aeruginosa*^#6)^   CTRX (CTX) or SBT/ABPC   - Risk of *P. aeruginosa*^#6)^   CFPM，TAZ/PIPC，or MEPM (DRPM, IPM/CS) ^#5)^  Consider addition of the following agents: VCM (or LZD) and AMK^#7)^ | ◆ Prescription of anti-MRSA agents should be limited to a situation where MRSA is of concern.^#8)^  ◆ Addition of aminoglycoside is controversial. Special caution is required when an aminoglycoside is given with vancomycin. |
| Community-acquired urinary tract infection | Mostly *E. coli* | ABPC plus GM  or  CTRX (CTX) |  |
| Catheter-associated or health care–associated urinary tract infection | *E. coli*, *P. aeruginosa*, *Enterococcus* spp. | TAZ/PIPC，MEPM (DRPM, IPM/CS)^#5)^ or CPFX  Consider addition of GM or AMK | GNR including *P. aeruginosa* should be treated. |
| Catheter-related bloodstream infection | *Staphylococcus epidermidis*, *S. aureus* (including MRSA), nosocomial GNB such as *P. aeruginosa* | VCM  plus  CFPM，TAZ/PIPC，or MEPM (DRPM, IPM/CS)^#5)^  Consider addition of the following agents  GM or AMK  FLCZ or MCFG |  |
| Community-onset noncomplicated intra-abdominal infection | Anaerobes such as *Bacteroides* spp., susceptible GNB such as *E. coli* | APBC/SBT | TAZ/PIPC is appropriate where *P. aeruginosa* is of concern. |
| Complicated intra-abdominal infection | In addition to all of the above  Nosocomial GNB such as *P. aeruginosa* | TAZ/PIPC or MEPM (DRPM, IPM/CS)^#5)^  Consider addition of the following agents:  VCM if MRSA is of concern  FLCZ or MCFG if *Candida* spp. is of concern |  |
| Complicated skin and soft tissue infection | - Community onset and without particular background history   *Streptococcus pyogenes*, MSSA, *Clostridium* spp.   - Exposure to seawater or freshwater   *Aeromonas hydrophila, Vibrio vulnificus*   - Diabetic foot, ischemic limb, health care–associated   *S. aureus*, nosocomial GNB such as *P. aeruginosa* | - Community onset and without particular background history   PCG plus CLDM   - Exposure to seawater or freshwater   MEPM (DRPM, IPM/CS)^#5)^ plus CPFX   - Diabetic foot, ischemic limb, health care–associated   TAZ/PIPC，MEPM (DRPM, IPM/CS)^#5)^  Consider addition of LZD if CA-MRSA is of concern | Consider addition of LZD where CA-MRSA is clinically or epidemiologically of concern. |
| Community-acquired meningitis | *S. pneumoniae, Neisseria meningitidis* | High-dose CTRX (2gQ12h) (high-dose CTX 2gQ4h)  plus  High-dose VCM (20mg/kgQ12h)  plus  aciclovir | ABPC (2gQ4h) should be added to treat *Listeria monocytogenes* for patients age $\geq$50 years and/or with alcoholism.  Dexamethasone (0.15 mg/kg) needs to be given before any antibiotics and to be continued for 2–4 days. |
| Post-neurosurgical meningitis | *S. aureus* including MRSA, nosocomial GNB such as *P. aeruginosa* | High-dose VCM (20mg/kgQ12h)  plus  High-dose CFPM (2gQ8h) or high-dose MEPM (2gQ8h) |  |
| Febrile neutropenia | Nosocomial GNB, especially *P. aeruginosa, S. aureus* including MRSA | CFPM，TAZ/PIPC，or MEPM (DRPM, IPM/CS)^#5)^  plus  VCM  Consider addition of GM or AMK |  |
| Community-onset sepsis with unknown source of infection | *S. pneumoniae*, *N. meningitidis*, susceptible GNB such as *E. coli* | - Meningitis is likely   High-dose CTRX (2gQ12h) (or high-dose CTX 2gQ4h)  plus  High-dose VCM (20mg/kgQ12h)  plus  acyclovir  plus  GM (7 mg/kg single dose)   - Meningitis is unlikely   CTRX (CTX)  plus  GM (7 mg/kg single dose) | Infectious Disease (ID) specialist consultation is required.  Where meningitis is likely, ABPC (2gQ4h) should be added to treat *L. monocytogenes* for patients age $\geq$50 years and/or with alcoholism. |
| Nosocomial (nursing and health care–associated) sepsis with unknown source of infection | Nosocomial GNB such as *P. aeruginosa*, *S. aureus* including MRSA | CFPM，TAZ/PIPC，or MEPM (DRPM, IPM/CS)^#5)^  plus  VCM  Consider addition of AMK | ID specialist consultation is required. |

1. These recommendations are for severe sepsis and septic shock. Therefore, they may not be applicable to other situations.
2. Abbreviated names of antibiotics are based on the Japanese Society of Chemotherapy glossary.
3. In these recommendations, only those drugs with internationally rich clinical evidences and profound clinical trials are listed. However, depending on drug availability at each institution, similar drugs in the same class may be chosen.
4. Risk factors for *P. aeruginosa* infection in community-acquired pneumonia [54]： (i) long-term use of steroid, (ii) chronic severe respiratory diseases (e.g., chronic obstructive lung disease, asthma), (iii) alcoholism, and (iv) frequent exposure to antibiotics
5. As a general rule，prescription of carbapenems (MEPM，DRPM, IMP/CS) should be limited to situations where they are primarily indicated. The use of carbapenems is justified in the following situations: (i) history of being treated with both CFPM (CZOP or CPR) and TAZ/PIPC (including PIPC) in the past 3 months, (ii) unacceptably high resistance rate of *P. aeruginosa* to both CFPM (CZOP or CPR) and TAZ/PIPC (including PIPC) with preserved susceptibility to carbapenems in local antibiograms, (iii) endemicity of ESBL producers and *Acinetobacter baumannii*，and (iv) known colonization of GNB that are susceptible only to carbapenems (e.g., ESBL producers, *A. baumannii*).
6. Risk factors of *P. aeruginosa* infection [54]： (i) length of hospital stay $\geq$5 days, (ii) antibiotic exposure in the past 3 months, (iii) endemicity of resistant nosocomial GNB such as *P. aeruginosa*, (iv) immunocompromised status, (v) hospitalization$\geq$2 days in the past 3 months, and (vi) chronic hemodialysis
7. Where clinically acceptable susceptibilities of anti-*Pseudomonas* beta-lactams are not expected based on local epidemiology or patient’s background, addition of AMK (15 mg/kg, once daily) is an acceptable strategy. Prolonged use of aminoglycosides may cause renal failure. Thus, the use of aminoglycosides for this purpose should be limited to a single dose or, at the longest, for 3 days.
8. Risk factors of MRSA infection [54]： (i) recent prolonged use ($\geq$14 days) of antibiotics, (ii) known colonization of MRSA, (iii) cluster GPC and/or GPC phagocytosis in Gram staining of lower respiratory tract specimen.

**Table III-3-2. Definitive antibiotic therapy according to causative organisms^#1, 2)^**

| Causative organism | Antibiotic recommendation | Other options | Notes |
| --- | --- | --- | --- |
| Gram-positive cocci | | | |
| *Enterococcus* spp. | ·ABPC (2gQ4h)  ·VCM (if ABPC resistant) |  | If the MIC of GM is <500 mg/L，addition of GM (1mg/kgQ8h) needs to be considered.  ID consultation is required |
| *Staphylococcus aureus* (MSSA) | · CEZ (2gQ8h) |  | Where central nervous system is involved, CEZ cannot be chosen.  ID specialist consultation is required. |
| *Staphylococcus epidermidis* | ·VCM |  |  |
| *Streptococcus pneumonia* (penicillin susceptible) | ·PCG，ABPC | ·CTRX (CTX) |  |
| Group A, B, C, F, G Streptococci | ·PCG，ABPC | ·CEZ  ·CLDM |  |
| *Streptococcus viridans* | ·PCG，ABPC | ·CTRX (CTX) |  |
| Gram-positive bacilli | | | |
| *Bacillus anthracis* | ·PCG，ABPC | ·CPFX |  |
| *Corynebacterium jeikeium* | ·VCM | ·LZD |  |
| *Listeria monocytogenes* | ·ABPC |  |  |
| *Nocardia* spp. | ·ST |  | ID specialist consultation is required especially for central nervous infections. |
| Gram-negative cocci | | | |
| *Neisseria meningitidis* | ·PCG，ABPC | ·CTRX (CTX) |  |
| Gram-negative bacilli | | | |
| *Aeromonas hydrophila* | ·CPFX | ·MEPM (DRPM, IPM/CS)  ·CFPM |  |
| *Achromobacter xylosoxidans* | ·MEPM (DRPM, IPM/CS) | ·ST | ID specialist consultation is required. |
| *Acinetobacter baumannii* | ·MEPM (DRPM, IPM/CS) | ·High-dose SBT/ABPC (3gQ6h)  ·CPFX(400mgQ8h) | Accurate identification of *A. baumannii* at the hospital laboratory is generally unfeasible. |
| *Burkholderia cepacia* | ·ST |  | ID consultation is required. |
| *Campylobacter jejuni* | ·CPFX |  |  |
| *Citrobacter* spp. | ·CFPM | ·CPFX  ·MEPM (DRPM, IPM/CS) |  |
| *Escherichia coli* | ·ABPC  ·CEZ  ·CTRX |  | For ESBL producers, MEPM (DRPM, IPM/CS) is recommended. |
| *Enterobacter* spp. | ·CFPM | ·CPFX  ·MEPM (DRPM, IPM/CS) |  |
| *Haemophilus influenzae* | ·ABPC  ·CTRX (CTX) (if resistant to ABPC) |  |  |
| *Klebsiella* spp. | ·CEZ  ·CTRX (CTX) |  | For ESBL producers, MEPM (DRPM, IPM/CS) is recommended. |
| *Legionella pneumophila* | ·LVFX  ·AZM |  |  |
| *Moraxella catarrhalis* | ·CTRX (CTX)  ·SBT/ABPC |  |  |
| *Pasteurella multocida* | ·PCG，ABPC |  |  |
| *Proteus mirabilis* | ·ABPC  ·CEZ |  | For ESBL producers, MEPM (DRPM, IPM/CS) is recommended. |
| *Proteus vulgaris* | ·CTRX (CTX)  ·CFPM | ·CPFX |  |
| *Pseudomonas aeruginosa* | ·High-dose PIPC (4gQ4–6h)  ·TAZ/PIPC  ·High-dose CAZ (2gQ8h)  ·CFPM | ·MEPM (DRPM, IPM/CS)  ·CPFX | PIPC is equivalent to TAZ/PIPC in the treatment of *P. aeruginosa*.  MEPM，DRPM or IPM/CS should be chosen where the isolate is resistant to all other recommended antibiotics. |
| *Salmonella* spp. | ·CTRX (CTX) | ·CPFX |  |
| *Serratia marcescens* | ·CFPM | ·CPFX  ·MEPM (DRPM, IPM/CS) |  |
| *Shigella* spp. | ·CPFX | ·ST  ·AZM |  |
| *Stenotrophomonas maltophilia* | ·ST |  | If resistant to ST, ID specialist consultation is required. |
| *Vibrio cholera* | ·CPFX | ·ST |  |
| *Vibrio vulnificus* | ·MINO＋CAZ |  |  |
| Anaerobes | | | |
| *Actinomyces* spp. | ·ABPC | ·CLDM |  |
| *Bacteroides fragilis* group | ·SBT/ABPC  ·TAZ/PIPC | ·CLDM  ·CMZ  ·MEPM (DRPM, IPM/CS) |  |
| *Clostridium difficile* | ·Oral VCM |  |  |
| Non-*difficile Clostridium* spp. | ·PCG | ·CLDM | In necrotizing fasciitis, combination therapy of PCG plus CLDM is recommended. |
| *Fusobacterium nucleatum* | ·SBT/ABPC  ·CLDM |  |  |
| *Peptococcus* spp. | ·PCG，ABPC |  |  |
| *Peptostreptococcus* spp. | ·PCG，ABPC |  |  |
| *Prevotella* spp. | ·PCG，ABPC |  |  |
| Mycoplasma | | | |
| *Mycoplasma pneumoniae* | ·EM, AZM, CPFX, LVFX |  |  |
| Fungi | | | |
| *Candida albicans* | ·FLCZ |  |  |
| *Candida tropicalis* | ·FLCZ |  |  |
| *Candida parapsilosis* | ·FLCZ |  |  |
| *Candida glabrata* | ·MCFG |  |  |
| *Candida krusei* | ·MCFG |  |  |
| *Candida lusitaniae* | ·MCFG | ·AMPH-B，VRCZ |  |
| *Aspergillus fumigatus* | ·VRCZ | ·AMPH-B |  |
| *Aspergillus flavus* | ·VRCZ |  |  |
| *Aspergillus terreus* | ·VRCZ |  |  |

1. In these recommendations, only those drugs with internationally rich clinical evidences and profound clinical trials are listed. However, depending on drug availability at each institution, similar drugs in the same class may be chosen.
2. Refer to Q4 with regard to the treatment of resistant organism.

**Table III-3-3. Treatment option for highly drug-resistant and/or multidrug-resistant organisms**

| Organism | Antibiotic recommendation | Other options | Notes |
| --- | --- | --- | --- |
| Nosocomial MRSA | - VCM | - TEIC - LZD - DAP | - DAP is ineffective against pneumonia - VCM trough concentration should be maintained at 15-20 mg/L, with dose and interval adjustment. |
| CA-MRSA | - LZD - VCM plus CLDM |  |  |
| VISA, VRSA | - LZD - DAP |  | - DAP is ineffective against pneumonia. |
| Vancomycin-resistant *E. faecalis* | - High-dose ABPC (2gQ4h) (if susceptible to ABPC) - TEIC (if susceptible to TEIC) - LZD |  | - Generally resistant to QPR/DPR |
| Vancomycin-resistant *E. faecium* | - TEIC (If susceptible to TEIC） - LZD | - QPR/DPR |  |
| Penicillin-resistant *S. pneumoniae* (nonmeningitis) | - High-dose ABPC (2gQ4h) - High-dose CTRX (2gQ12h) - High-dose CTX (2gQ6h) | - LVFX |  |
| Penicillin intermediately resistant *S. pneumoniae* (meningitis) | - High-dose CTRX (2gQ12h) - High-dose CTX (2gQ6h) |  |  |
| Penicillin-resistant *S. pneumoniae* (meningitis) | - High-dose VCM (20mg/kgQ12h)   plus  High-dose CTRX (2gQ12h) or high-dose CTX (2gQ4h)  Consider addition of oral RFP |  | - VCM trough concentration should be targeted at 20 microgram/L. To achieve this as early as possible, the initial loading dose should be 30 mg/kg, then the second dose (20 mg/kg) should be given at 8 h after the initial dose. |
| Beta-lactamase nonproducing ampicillin resistant *H. influenzae* (BLNAR) (nonmeningitis) | - CTRX (1gQ12h) - CTX (1gQ６h) |  |  |
| Beta-lactamase nonproducing ampicillin resistant *H. influenzae* (BLNAR)(meningitis) | - High-dose CTRX (2gQ12h) - High-dose CTX (2gQ6h) | - High-dose MEPM (2gQ8h) |  |
| ESBL producers | - MEPM (DRPM, IPM/CS) | - AMK (15 mg/kg once daily, if susceptible to AMK, only for urinary tract infection) - CPFX (400mgQ8h, if susceptible to CPFX) |  |
| Carbapenem intermediately resistant Enterobacteriaceae | - CPFX (400mgQ8h, if susceptible to CPFX) - Extended infusion of high-dose MEPM (2gQ8h) - Colistin^#1)^ | - AMK (15 mg/kg once daily, if susceptible to AMK, only for urinary tract infection) | - Extended infusion: infusion over 3 h |
| Carbapenem-resistant Enterobacteriaceae | - Colistin^#1)^ | - AMK (15 mg/kg once daily, if susceptible to AMK, only for urinary tract infection) - CPFX (400mgQ8h, if susceptible to CPFX) | - Dose of colistin: colistin base 2.5–5.0 mg⋅kg^-1^⋅day^-1^ (colistimethate sodium 6.67–13.3 mg⋅kg^-1^⋅day^-1^), divided into 2–4 times a day |
| Pan-beta-lactam-resistant, fluoroquinolone-resistant *P. aeruginosa* | - Colistin^#1)^ | - AMK (15 mg/kg once daily, if susceptible to AMK, only for urinary tract infection) | - Dose of colistin: colistin base 2.5–5.0 mg⋅kg^-1^⋅day^-1^ (colistimethate sodium 6.67–13.3 mg⋅kg^-1^⋅day^-1^), divided into 2–4 times a day |
| Carbapenem intermediately resistant *A. baumannii* | - High-dose SBT/ABPC (3–4.5gQ6h, if susceptible to SBT/ABPC) - Extended infusion of high-dose MEPM (2gQ8h) - Colistin^#1)^ | - AMK (15 mg/kg once daily, if susceptible to AMK, only for urinary tract infection) - CPFX (400mgQ8h, if susceptible to CPFX) | - Extended infusion: infusion over 3 h |
| Carbapenem-resistant *A. baumannii* | - High-dose SBT/ABPC (3–4.5gQ6h, if susceptible to SBT/ABPC) - Colistin^#1)^ | - AMK (15 mg/kg once daily, if susceptible to AMK, only for urinary tract infection) - CPFX (400mgQ8h, if susceptible to CPFX) | - Dose of colistin: colistin base 2.5–5.0 mg⋅kg^-1^⋅day^-1^ (colistimethate sodium 6.67–13.3 mg⋅kg^-1^⋅day^-1^), divided into 2–4 times a day |

1. Colistin is not commercially available in Japan.
2. In these recommendations, only those drugs with internationally rich clinical evidences and profound clinical trials are listed. However, depending on drug availability at each institution, similar drugs in the same class may be chosen.

**Table III-3-4. PD parameters of representative classes of antibiotics that correlate with clinical efficacy**

| TAM | C_max_/MIC | AUC_0–24_/MIC |
| --- | --- | --- |
| Beta-lactams | Aminoglycosides | Aminoglycosides |
|  | Fluoroquinolones | Fluoroquinolones |
|  |  | Glycopeptides |

**Table III-3-5. Dose and intervals of representative antibiotics in product labeling in Japan and those recommended**

**from the PK/PD perspective ^#1)^**

| Antibiotics | Dose and intervals in product labeling in Japan (maximum dose) | Dose and intervals recommended based on PK/PD |
| --- | --- | --- |
| CEZ | 5 g (daily maximum dose) | 2gQ8h |
| CTX | 1gQ6h | 2gQ4h |
| CTRX | 1gQ12h | 1gQ12h |
| CAZ | 2gQ12h | 2gQ8h |
| CFPM | 2gQ12h | 2gQ8–12h |
| PCG | 4 million units Q4h | 4 million units Q4h |
| ABPC | 2gQ12h | 1gQ6h |
| SBT/ABPC | 3gQ12h | 1.5gQ6h |
| PIPC | 8 g (daily maximum dose) | 4gQ4–6h |
| TAZ/PIPC | 4.5gQ6h | 4.5gQ6–8h |
| IPM/CS | 0.5gQ6h | 1gQ8h |
| MEPM | 1gQ8h | 1gQ8h |
| DRPM | 1gQ8h | 500mgQ8h |
| GM | 60mgQ12h | 7 mg/kg once daily |
| AMK | 200mgQ12h | 15 mg/kg once daily |
| CPFX | 300mgQ12h | 400mgQ8h |
| LVFX | 500 mg once daily | 500–750 mg once daily |
| VCM | 1gQ12h | Initial loading dose of 25–30 mg/kg, then 15–20mg/kgQ8–12h，with TDM |

1. The recommended dose according to PK/PD is based on the assumption that patients with severe sepsis or septic shock have a creatinine clearance of >50 mL/min and no central nervous system infections. Doses of antipseudomonal agents are given in the table.

**Table III-3-6. Standard duration of antibiotic therapy for representative infectious diseases**

| Type of infectious diseases | Standard duration of antibiotic therapy (days) |
| --- | --- |
| Community-acquired pneumonia | $\geq$5 |
| Ventilator-associated pneumonia, hospital-acquired pneumonia, and nursing and health care–associated pneumonia |  |
| Organisms other than nonfermenters | 7 |
| Nonfermenters | 14 |
| Complicated urinary tract infections | 14 |
| Bacterial meningitis |  |
| *Neisseria meningitides* | 7 |
| *Haemophilus influenzae* | 7 |
| *Streptococcus pneumoniae* | 10–14 |
| GNB | 21 |
| *Listeria monocytogenes* | 21 |
| Intra-abdominal infections | 4–7 after source control |
| Bloodstream infections including CRBSI |  |
| *Staphylococcus epidermidis* | 5–7 |
| *Staphylococcus aureus* | $\geq$14 |
| *Enterococcus* spp. | 7–14 |
| GNB | 7–14 |
| *Candida* spp. | 14 after the first negative blood culture |

**Table III-4-1**

|  | X ray | Ultrasound | CT | MRI |
| --- | --- | --- | --- | --- |
| Meningitis/encephalitis | 〇 |  | ◎ | FLAIR, enhanced T1 |
| Cervical abscess, soft tissue infection | ◎ | 〇 | Enhanced CT ◎, MDCT 〇 | T2 |
|  |  |  |  |  |
| Respiratory infection | Chest ◎ |  | Plain chest ◎, HRCT |  |
| Biliary infection | Abdomen ◎ | ◎ | Enhanced abdominal CT ◎ | MRCP |
| Urinary tract infection | KUB | ◎ | Enhanced abdominal/pelvic CT ◎，MDCT |  |
| Septic emboli | Chest ◎ | Cardiac ◎,  cervical veins 〇 | Plain chest ◎ |  |

◎: Most recommended imaging studies

〇: Secondarily recommended imaging studies

Abbreviations: FLAIR, fluid attenuated inversion recovery; MDCT, multidetector row CT; HRCT, high-resolution CT; KUB, kidney-ureter-bladder; MRCP, magnetic resonance cholangiopancreatography


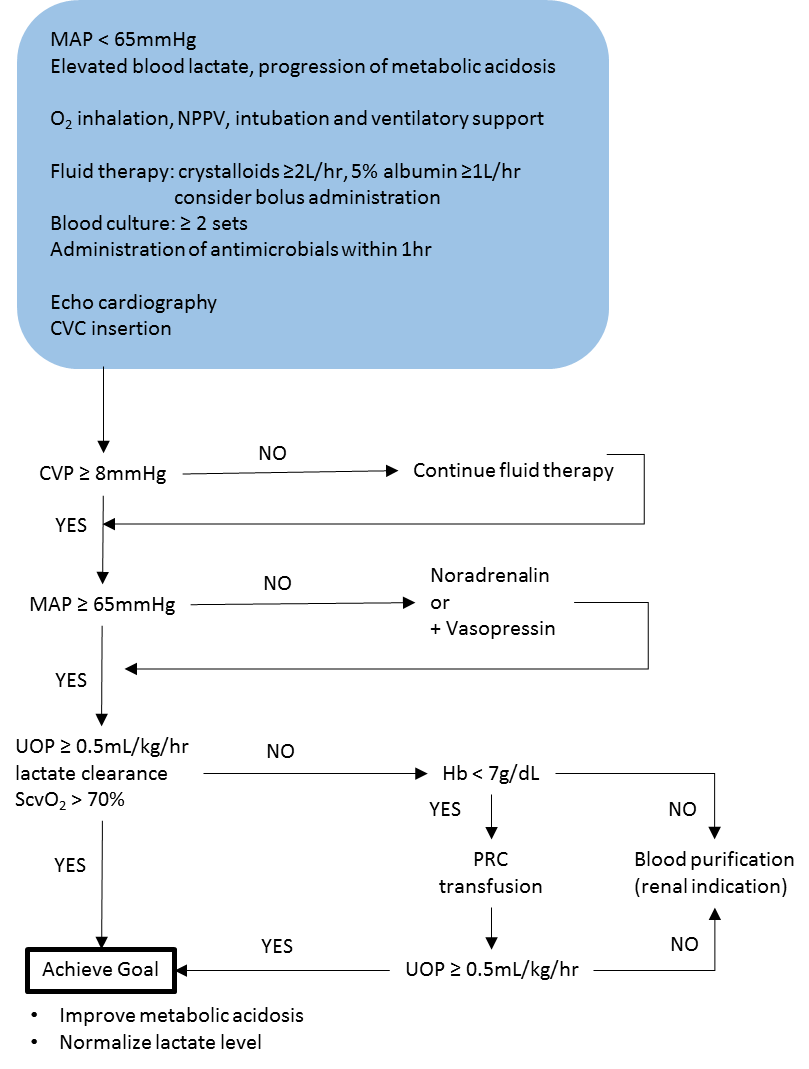


**Fig IV-5-1. Example of initial resuscitation**

**Table IV-6-1**

| Author/journal | | Amato/*NEJM* 1998 [141] | Brochard/*AJRCCM* 1998 [142] | Brower/*CCM* 1999 [143] | Stewart/*NEJM* 1998 [144] | ARDSNet/*NEJM* 2000 [145] |
| --- | --- | --- | --- | --- | --- | --- |
| Control | N | 24 | 58 | 26 | 60 | 429 |
|  | VT | 12.0 | 10.3 (1.7) | 10.2 (0.1) | 10.8 (1.0) | 11.8 (0.8) |
|  | Pplat | 34.4 (1.9) | 31.7 (6.6) | 30.6 (0.8) | 26.8 (6.7) | 33 (9) |
|  | Mortality | 71.0% | 37.9% | 46.0% | 47.0% | 39.8% |
| Protective | N | 29 | 58 | 26 | 60 | 432 |
|  | VT | 6.0 | 7.1 (1.3) | 7.3 (0.1) | 7.2 (0.8) | 6.2 (0.9) |
|  | Pplat | 31.8 (1..4) | 25.7 (5.0) | 24.9 (0.8) | 22.3 (5.4) | 25 (7) |
|  | Mortality | 38.0%* | 46.6% | 50.0% | 50.0% | 31.0%* |

Note: For five RCTs that investigated the effect of tidal volume on the prognosis of ALI/ARDS, the number of cases, actual tidal volume (converted to a standard body weight equivalent), plateau pressure, and mortality are shown. Although a significant difference in mortality was found in only two studies (Amato and ARDSNet), the mortality of the control group is significantly higher in the meta-analysis because the number of cases of the ARDSNet study was considerably large. Pay attention to the difference between the plateau pressure in CQ2. The number in parenthesis ( ) shows 1 standard deviation.

*: Statistically significant.

ALI/ARDS, acute lung injury/acute respiratory distress syndrome; RCT, randomized controlled trial; SD, standard deviation; VT, tidal volume.
